# Optimizing PD-1/PD-L1 inhibitors plus chemotherapy for driver mutation–negative non-squamous NSCLC in China: a cost-effectiveness analysis

**DOI:** 10.3389/fphar.2026.1849145

**Published:** 2026-08-21

**Authors:** Jianwei Ji, Qian Du, Mengmeng Teng, Fuhong Qin, Ruikang Zhang, Yalin Dong

**Affiliations:** 1 Department of Pharmacy, The First Affiliated Hospital of Xi’an Jiaotong University, Xi’an, Shannxi, China; 2 Department of Pharmacy, The Second Affiliated Hospital of Zhengzhou University, Zhengzhou, Henan, China

**Keywords:** cost-effectiveness, markov model, non-squamous NSCLC, PD-1/PD-L1 inhibitors, toripalimab

## Abstract

**Background:**

PD-1/PD-L1 inhibitors combined with chemotherapy have improved survival outcomes in patients with driver mutation–negative non-squamous non-small cell lung cancer (non-sqNSCLC). However, the comparative efficacy, safety, and economic value of available PD-1/PD-L1 inhibitor–based regimens remain uncertain in China.

**Methods:**

We conducted a network meta-analysis to indirectly compare the efficacy and safety of 14 first-line treatment regimens, including 13 PD-1/PD-L1 inhibitor–based chemotherapy regimens and chemotherapy alone, in patients with driver mutation–negative non-sqNSCLC. Clinical evidence was subsequently integrated into a Markov model with a 10-year time horizon and 3-week cycles to evaluate cost-effectiveness from the perspective of the Chinese healthcare system. Transition probabilities were derived from reconstructed survival curves using parametric survival models. The primary economic outcomes included total costs, quality-adjusted life-years (QALYs), incremental cost-effectiveness ratios (ICERs), and net monetary benefits (NMBs). Deterministic and probabilistic sensitivity analyses were performed to assess model robustness.

**Results:**

The network meta-analysis demonstrated that toripalimab, sintilimab, and pembrolizumab combined with chemotherapy provided superior efficacy and favorable safety among 13 PD-1/PD-L1 inhibitors with chemotherapy for non-sqNSCLC. In the cost-effectiveness analysis, toripalimab with chemotherapy achieved 2.583 QALYs at a total cost of $39,232.33. And it was cost-effective compared with chemotherapy alone, camrelizumab-, tislelizumab-, and sintilimab-based chemotherapy regimens, with ICERs ranging from $10,624.47 to $16,120.12 per QALY, all below the willingness-to-pay threshold of $41,859/QALY. Compared with chemotherapy alone, camrelizumab, tislelizumab, sintilimab with chemotherapy were cost-effective, with ICERs of $28,813.78/QALY, $19,278.47/QALY, and $20,315.01/QALY, respectively. PFS and PD utilities were key drivers of cost-effectiveness, while drug costs predominantly influenced regimens with ICERs.

**Conclusion:**

Among currently available first-line treatment strategies for driver mutation–negative non-sqNSCLC in China, toripalimab and sintilimab combined with chemotherapy demonstrated favorable clinical and economic profiles. These findings provide evidence to support treatment selection, reimbursement decisions, and healthcare resource allocation in clinical practice.

## 1 Introduction

Lung cancer remains the leading cause of cancer-related morbidity and mortality globally, with non-small cell lung cancer (NSCLC) representing approximately 85% of all lung cancer cases (Bray et al., 2024). Among NSCLC, non-squamous NSCLC (non-sqNSCLC) accounts for approximately 70% of cases (Duma et al., 2019). At the time of diagnosis, most patients present with advanced or metastatic disease, for which chemotherapy has historically been the standard treatment (Singh et al., 2022). However, the survival benefits achieved with chemotherapy alone remain limited. In recent years, the introduction of targeted therapy and immunotherapy has significantly improved survival outcomes in patient with NSCLC (Florez et al., 2025). Nevertheless, genetic mutations are detected in only about 30% of patients with non-sqNSCLC, limiting the applicability of targeted therapies (Qian et al., 2025). Consequently, immunotherapy has emerged as the standard first-line treatment for patients without driver mutations, as well as for those who develop resistance following targeted therapy.

Current clinical practice guidelines, including those issued by the Chinese Society of Clinical Oncology and the American Society of Clinical Oncology, recommend programmed death protein 1 (PD-1)/programmed death-ligand 1 (PD-L1) inhibitors combined with chemotherapy as the preferred first-line treatment for patients with advanced, driver mutation–negative non-sqNSCLC (NIH, 2025; NCCN, 2026). This treatment strategy is also recommended for patients with driver mutations who have progressed on tyrosine kinase inhibitor therapy. To date, more than 20 PD-1/PD-L1 inhibitors have been approved worldwide, with over 10 agents for first-line treatment of non-sqNSCLC (NIH, 2025; Mizuno et al., 2022; Mountzios, 2025). Compared with chemotherapy alone, PD-1/PD-L1 inhibitors with chemotherapy significantly improve both overall survival (OS) and progression-free survival (PFS), although these benefits are accompanied by an increased risk of adverse events (AEs) (Chen et al., 2025; Rao et al., 2025). However, direct head-to-head comparisons among different PD-1/PD-L1 inhibitors–based regimens are lacking, resulting in uncertainty regarding relative efficacy and safety. Although several meta-analyses have evaluated these therapies, contemporary evidence specifically focusing on patients with driver mutation–negative non-sqNSCLC remains limited, particularly with the recent approval of PD-1/PD-L1 inhibitors.

Beyond clinical effectiveness, the high cost of PD-1/PD-L1 inhibitors has become a substantial economic challenge for healthcare systems worldwide. Global sales of PD-1/PD-L1 inhibitors generated $52 billion in 2023, and are projected to exceed $90 billion by 2028 (Tay-Teo et al., 2025). In China, substantial differences in drug acquisition costs, reimbursement policies, and national centralized procurement have resulted in considerable variation in the affordability and accessibility of individual PD-1/PD-L1 inhibitors (Yi et al., 2025; Zhou J. et al., 2024). Furthermore, emerging PD-1/PD-L1 inhibitors, such as sugemalimab and serplulimab, are being increasingly adopted into clinical practice. Although these newer agents have demonstrated encouraging clinical efficacy, robust comparative evidence regarding their relative effectiveness, safety, and economic value remains limited. Previous economic evaluations have primarily focused on individual PD-1/PD-L1 inhibitor–based regimens using data from single randomized controlled trials (RCTs) and therefore cannot adequately inform comparisons across multiple treatment strategies (Jiang and Wang, 2022; Lu T. T. et al., 2023; Peng et al., 2024). Consequently, comprehensive evidence integrating comparative efficacy, safety, and cost-effectiveness is urgently needed to support evidence-based clinical decision-making, reimbursement policy, and rational healthcare resource allocation.

In this context, we conducted a systematic review and network meta-analysis to assess the efficacy and safety of PD-1/PD-L1 inhibitor–based chemotherapy regimens in driver mutation–negative non-sqNSCLC. In addition, a cost-effectiveness analysis was performed to evaluate the relative economic value of currently available regimens. The findings aim to inform clinical decision-making and guide formulary selection by identifying treatments that are safe, effective, and economically sustainable.

## 2 Methods

### 2.1 Literature search

The systematic review was conducted in accordance with the predefined PICOS framework (Supplementary Table S1). A comprehensive literature search was performed in PubMed, Embase, Web of Science, and the Cochrane Library from database inception to 10 December 2025, with an updated search conducted on 1 July 2026. The detailed search strategy for PubMed is provided in Supplementary Table S2. The search strategy combined controlled vocabulary terms (e.g., MeSH) and free-text keywords related to immune checkpoint inhibitors, PD-1/PD-L1 inhibitors, non-small cell lung cancer, non-squamous histology, and randomized controlled trials. Studies were eligible for inclusion if they met the following criteria: (1) patients with histologically or pathologically confirmed driver mutation-negative non-sqNSCLC; (2)first-line treatment consisting of a PD-1/PD-L1 inhibitor combined with chemotherapy, with chemotherapy alone as the comparator; (3) reporting at least PFS or OS outcomes. Exclusion criteria included: (1) participants younger than 18 years; (2) incomplete data or duplicated reports, (3) studies restricted to patients with specific PD-L1 expression levels or tumor proportion score categories. Screening was conducted by DQ, TMM, and ZRK, sequentially based on titles, abstracts, and full texts, resulting in the final selection of eligible studies. Two reviewers independently extracted study characteristics, including study design, publication year, patient characteristics, PFS, OS, AEs, grade ≥3 AEs (sAEs). The study protocol was prospectively registered in the International Prospective Register of Systematic Reviews (PROSPERO; registration number CRD420251252371). As this study was based exclusively on published aggregate data and did not involve identifiable individual patient information, ethical approval and informed consent were not required.

### 2.2 Network meta-analysis

A network meta-analysis (NMA) was performed using R version 4.3.3 software with “*gemtc*” package to compare the relative efficacy and safety in PD-1/PD-L1 inhibitors with chemotherapy indirectly under a fixed-effect or random effect model. The assessed treatment strategies included pembrolizumab with chemotherapy (Pemb-Chem), nivolumab with chemotherapy (Nivo-Chem), atezolizumab with chemotherapy (Atez-Chem), durvalumab with chemotherapy (Durv-Chem), camrelizumab with chemotherapy (Camr-Chem), cemiplimab with chemotherapy (Cemi-Chem), retifanlimab with chemotherapy (Reti-Chem), sintilimab with chemotherapy (Sint-Chem), serplulimab with chemotherapy (Serp-Chem), sugemalimab with chemotherapy (Suge-Chem), tislelizumab with chemotherapy (Tisl-Chem), toripalimab with chemotherapy (Tori-Chem), prolgolimab with chemotherapy (Prol-Chem), and chemotherapy alone. By integrating both direct and indirect evidence within a connected treatment network, the relative efficacy of each regimen was evaluated in terms of PFS, OS, and objective response rate (ORR), while safety outcomes included AEs, sAEs and discontinuation because of AEs. Network inconsistency and publication bias were assessed, and the corresponding results are presented in Supplementary Table S3. The surface under the cumulative ranking curve (SUCRA) was calculated to estimate the ranking probabilities of each treatment in efficacy and safety outcomes.

### 2.3 Economic evaluation model

**FIGURE 1:**
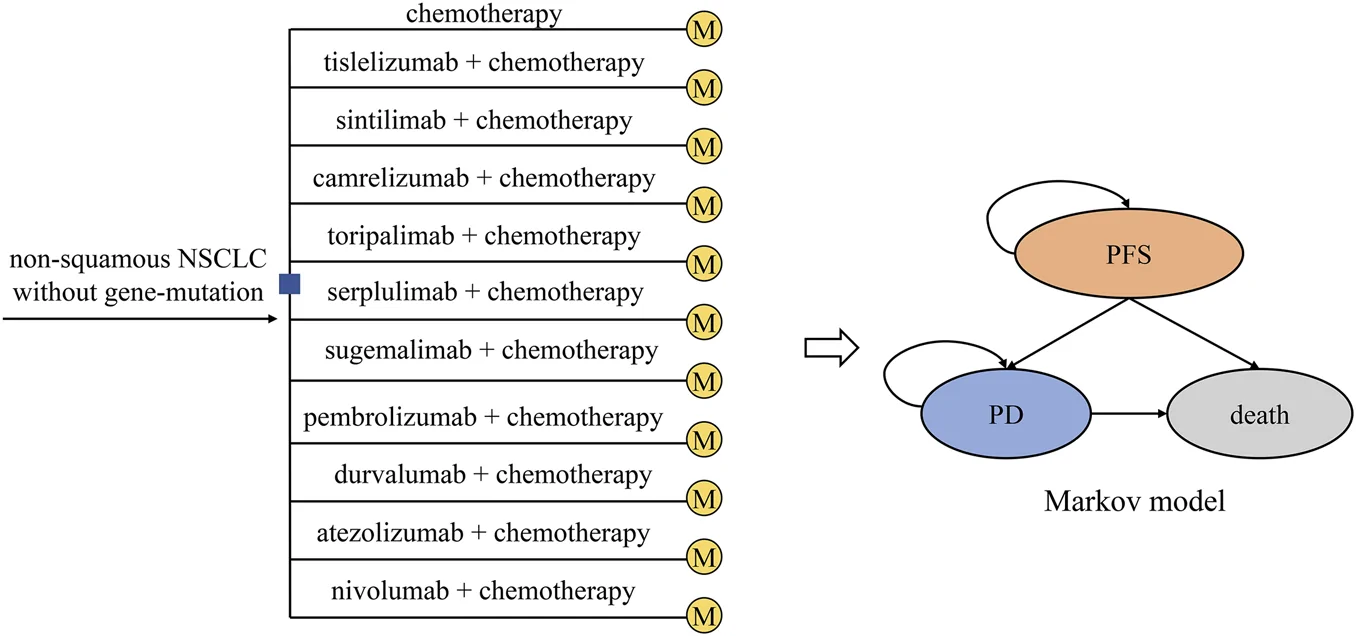
Health state transitions in Markov model. PFS, progression-free survival; PD, progression disease.

A Markov model was constructed in R version 4.3.3 software (“*heemod*” package) to evaluate the cost-effectiveness of 11 mutually exclusive treatment strategies (Figure 1): including Pemb-Chem, Nivo-Chem, Atez-Chem, Durv-Chem, Camr-Chem, Sint-Chem, Serp-Chem, Suge-Chem, Tisl-Chem, Tori-Chem, and chemotherapy alone. Cemiplimab, prolgolimab, and retifanlimab were excluded due to unavailability in China. Across all immunotherapy strategies, PD-1/PD-L1 inhibitors were discontinued after 2 years in patients who remained progression-free, consistent with the corresponding clinical trial protocols. The Markov model consisted of three health states: PFS, progressive disease (PD), and death. All patients entered the model in the PFS state and transitioned between health states according to the estimated transition probabilities. Patients in the PFS state could either remain in the PFS state, progress to the PD state, or die. Background mortality was applied to patients remaining in the PFS state. Patients in the PD state could either remain in PD or transition to death. Considering that patients would receive subsequent therapy after PD, we restricted the occurrence of sAEs to the PFS state. The model adopted a cycle length of 3 weeks, corresponding to the routine dosing interval, with a 10-year time horizon. The reporting of this study conforms to the Consolidated Health Economic Evaluation Reporting Standards (CHEERS 2022) statement (Supplementary Table S4).

### 2.4 Transition probabilities

Transition probabilities among the PFS, PD, and death states were estimated by reconstructing individual patient data (IPD) from published Kaplan–Meier survival curves and extrapolating survival beyond the observed follow-up period to 10 years (Guyot et al., 2012). For each treatment strategy, the most mature survival data available from published articles or conference presentations were used. Reconstructed IPD are presented in Supplementary Figures S1,S2. Parametric survival models, including Exponential, Weibull, log-normal, log-logistic, gamma, and Gompertz distributions, were fitted using the “*IPDfromKM*” package in R4.3.3. For the Atez-Chem strategy, survival curves were reconstructed by combining data from the IMpower130 and IMpower132 trials, whereas chemotherapy survival data were reconstructed using pooled data from all eligible studies. The optimal survival distribution was selected based on the Akaike information criterion and Bayesian information criterion (Supplementary Table S5; Supplementary Figure S3). The final parametric equations for PFS and OS are presented in Supplementary Table S6. Transition probabilities for each 3-week cycle were subsequently derived from the fitted PFS and OS functions.

### 2.5 Cost-effectiveness analysis

This study was conducted from the perspective of the Chinese healthcare system. Only direct medical costs were considered, including drug acquisition, administration, hospitalization, monitoring, post-progression treatment, best supportive care, and management of sAEs, based on local pricing and published literature (Table 1) (Shen et al., 2018; Lang et al., 2025; Peng et al., 2024; Wen et al., 2025). Treatment schedules and dosing assumptions were based on the RCTs and current clinical practice (Supplementary Table S7). For Atez-Chem, chemotherapy included pemetrexed with cisplatin or carboplatin and nab-paclitaxel with carboplatin. For Durv-Chem, patients received four cycles of induction therapy followed by maintenance durvalumab administered every 4 weeks. Health utility values for the PFS and PD health states were obtained from published studies (Shen et al., 2018; Lang et al., 2025). The incidence of hematological toxicities and sAEs with an incidence of at least 5% was extracted from the RCTs to estimate sAEs management costs and associated utility decrements for each regimen (Supplementary Table S8). Owing to the unavailability of detailed AEs data for the IMpower132, the cost and disutility of regimens estimates from the Chinese subgroup were used to calculate (Lu S. et al., 2023). Costs and utilities were discounted at an annual rate of 5%, with sensitivity analysis varying between 0% and 8%. A half-cycle correction was applied throughout the model.

**TABLE 1:** Summary of variables used as inputs in the cost-effectiveness model analysis.

Variable	Value	Range	Distribution	References
Clinical parameters				
Survival model for OS and PFS	Supplementary Table S7			RCTs
Risk for sAEs	Supplementary Table S8			RCTs
Health utility				
Utility of PFS	0.856	0.721–0.991	Beta	Shen et al. (2018)
Utility of PD	0.580	0.435–0.725	Beta	Lang et al. (2025)
Disutility of sAEs	Supplementary Table S8			Peng et al. (2024)
Cost ($)				
Serplulimab (100 mg)	513.89	411.11–616.67	gamma	local
Sintilimab (100 mg)	150.00	120.00–180.00	gamma	local
Pembrolizumab (200 mg)	2,488.61	1,990.89–2,986.33	gamma	local
Camrelizumab (200 mg)	357.87	286.29–429.44	gamma	local
Tislelizumab (100 mg)	174.10	139.28–208.92	gamma	local
Atezolizumab (1,200 mg)	4,555.56	3,644.44–5,466.67	gamma	local
Nivolumab (40 mg)	499.02	399.22–598.83	gamma	local
Sugemalimab (600 mg)	1,718.75	1,375.00–2,062.50	gamma	local
Toripalimab (240 mg)	261.79	209.43–314.14	gamma	local
Durvalumab (1,500 mg)	2,512.11	2,009.69–3,014.53	gamma	local
Pemetrexed (100 mg)	19.48	15.58–23.37	gamma	local
Cisplatin (10 mg)	1.94	1.55–2.33	gamma	local
Carboplatin (50 mg)	5.90	4.72–7.08	gamma	local
Nab-paclitaxel (30 mg)	18.34	14.67–22.00	gamma	local
Follow-up testing cost (per cycle)	51.97	41.58–62.36	Gamma	Peng et al. (2024)
Subsequent therapy cost (per cycle)	108.36	86.69–130.03	Gamma	Peng et al. (2024)
Best supportive care (per cycle)	315.70	252.56–378.84	Gamma	Peng et al. (2024)
Terminal care per patient	2,456.26	1,965.01–2,947.51	Gamma	Peng et al. (2024)
Discount rate	0.05	0–0.08	Uniform	​
Creatinine clearance rate (mL/min)	80	64–96	Normal	Wen et al. (2025)
Body surface area (m^2^)	1.72	1.38–2.06	Normal	Wen et al. (2025)
Mean weight (kg)	64	51.2–76.8	Normal	Wen et al. (2025)

PFS, progression-free survival; PD, progression disease; sAEs, grade ≥3 adverse events; RCTs, randomized controlled trials.

Total costs, quality-adjusted life-years (QALYs), and life-years (LYs) were calculated under willingness-to-pay threshold (WTP, $41,859, 3 times 2025 *per capita* GDP in China) over the 10-year time horizon. Incremental cost-effectiveness ratios (ICERs) and net monetary benefit (NMB) were computed to compare economic efficiency across regimens. The ICER was defined as the additional cost required to gain one additional QALY or LY compared with the reference strategy. An ICER below the WTP threshold indicates that the additional health benefits are achieved at an acceptable additional cost and that the intervention is considered cost-effective. NMB was calculated as NMB = QALYs × WTP − Cost, with NMB >0 indicating that the regimen provides economic benefit under the current WTP threshold.

### 2.6 Sensitivity analyses

Deterministic sensitivity analysis (DSA) and probabilistic sensitivity analysis (PSA) were conducted to evaluate the robustness of the base-case results. DSA assessed the impact of individual parameters on ICER compared with chemotherapy, and tornado diagrams were used for visualization. PSA was performed via 1,000 Monte Carlo simulations to by simultaneously sampling all model parameters from their respective probability distributions at a range of 0–10 times the GDP *per capita* ($13,953). Cost-effectiveness scatter plots were generated to illustrate the uncertainty in incremental costs and QALYs compared with chemotherapy, with the proportion of simulations below the WTP threshold representing the probability that each treatment strategy would be cost-effective. Cost-effectiveness acceptability curves were generated to estimate the probability that each treatment strategy would achieve the highest NMB across a range of WTP thresholds.

### 2.7 Statistical analysis

All statistical and economic analyses were performed using R version 4.4.3 and Microsoft Excel 2019. A two-sided *P* < 0.05 was considered statistically significant.

## 3 Results

### 3.1 Efficacy and safety analysis

**FIGURE 2:**
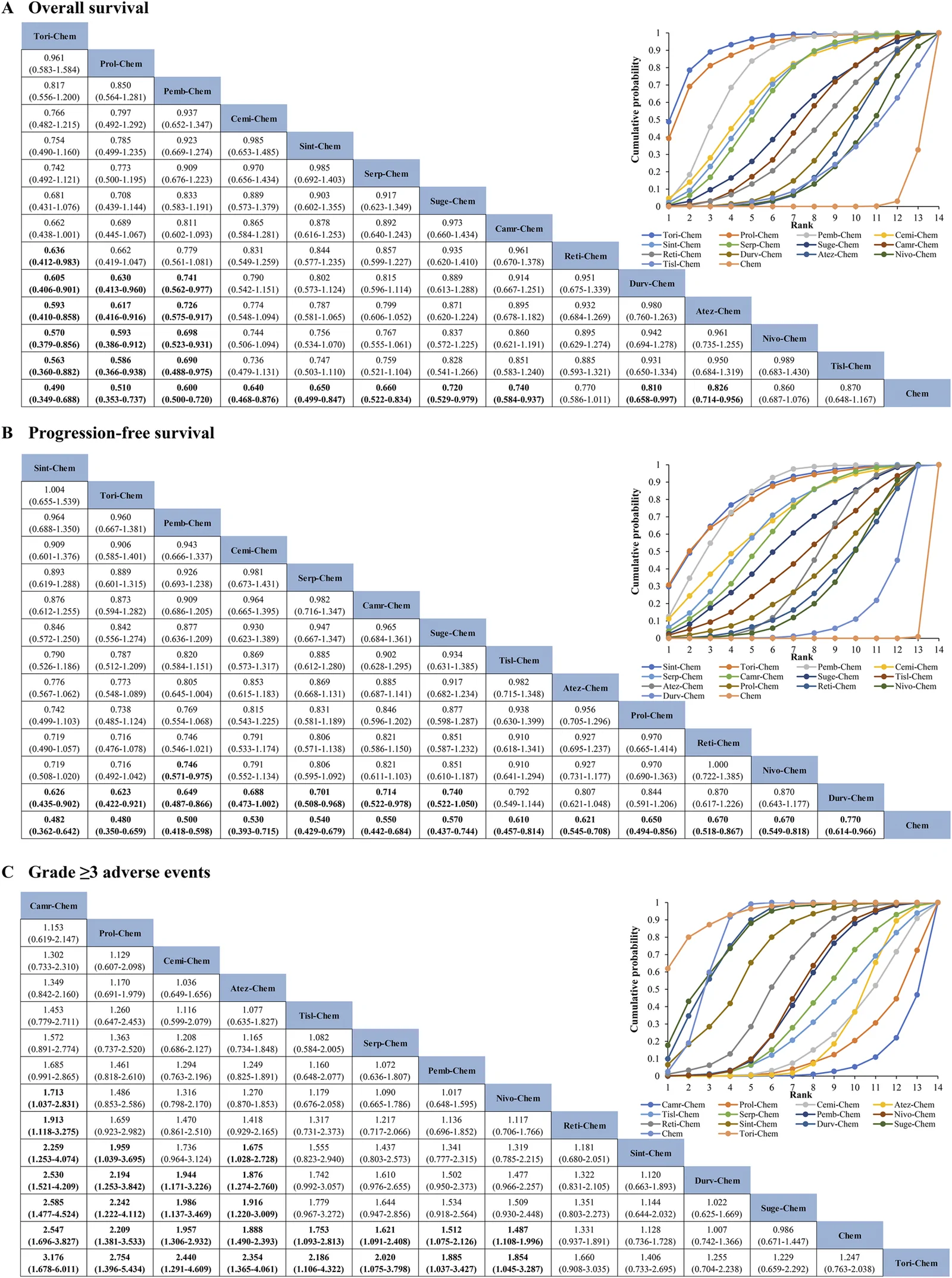
The efficacy and safety in Network Meta-analysis. **(A)** Overall survival; **(B)** progression-free survival; **(C)** grade ≥3 adverse events. Tori-Chem, toripalimab with chemotherapy; Pemb-Chem, pembrolizumab with chemotherapy; Cemi-Chem, cemiplimab with chemotherapy; Sint-Chem, sintilimab with chemotherapy; Serp-Chem, serplulimab with chemotherapy; Suge-Chem, sugemalimab with chemotherapy; Camr-Chem, camrelizumab with chemotherapy; Reti-Chem, retifanlimab with chemotherapy; Durv-Chem, durvalumab with chemotherapy; Atez-Chem, atezolizumab with chemotherapy; Nivo-Chem, nivolumab with chemotherapy; Tisl-Chem, tislelizumab with chemotherapy; Serp-Chem, serplulimab with chemotherapy; Prol-Chem, prolgolimab with chemotherapy; Chem, chemotherapy.

After study screening according to the predefined eligibility criteria, a total of 14 RCTs were included in the analysis (Zhou et al., 2025; Zhou et al., 2023a; Zhou et al., 2022; Zhou C. et al., 2024; Zhou et al., 2023b; Zhou et al., 2021; Zhong et al., 2024; Wang et al., 2023; Zhang et al., 2022; Yang et al., 2020; West et al., 2019; Nishio et al., 2021; Peters et al., 2025; Johnson et al., 2023; Makharadze et al., 2023; Gogishvili et al., 2022; Lu et al., 2025; Lu et al., 2024; Lu et al., 2021; Garassino et al., 2023; Gadgeel et al., 2020; Gandhi et al., 2018; Borghaei et al., 2023; Hao et al., 2026; Wang et al., 2025; Laktionov et al., 2025) (Supplementary Figure S4). Fourteen treatment strategies, including chemotherapy alone and 13 PD-1/PD-L1 inhibitor–based combination regimens, were included in the NMA. The characteristics of the included studies are summarized in Supplementary Table S9. The NMA results are presented for OS (Figure 2A), PFS (Figure 2B), objective response rate (ORR; Supplementary Figure S5A), AEs (Supplementary Figure S5B), sAEs (Figure 2C), and treatment discontinuation due to AEs (Supplementary Figure S5C). For OS, the Tori-Chem regimen was associated with a significantly lower risk of death than several other treatment strategies. In terms of PFS, all PD-1/PD-L1 inhibitors with chemotherapy significantly prolonged PFS compared with chemotherapy alone (*P* < 0.05). Regarding safety (Figure 2C), Camr-Chem was associated with the highest incidence of sAEs, whereas chemotherapy alone and Tori-Chem demonstrated comparatively lower risks of sAEs. Based on SUCRA rankings integrating both efficacy and safety, Tori-Chem, Sint-Chem, and Pemb-Chem demonstrated superior overall performance in terms of efficacy and safety in patients with non-sqNSCLC (Supplementary Table S10). Collectively, these findings indicate that Tori-Chem, Sint-Chem, and Pemb-Chem provided the most favorable balance between efficacy and safety among the evaluated treatment strategies.

### 3.2 Base-case cost-effectiveness analysis

**FIGURE 3:**
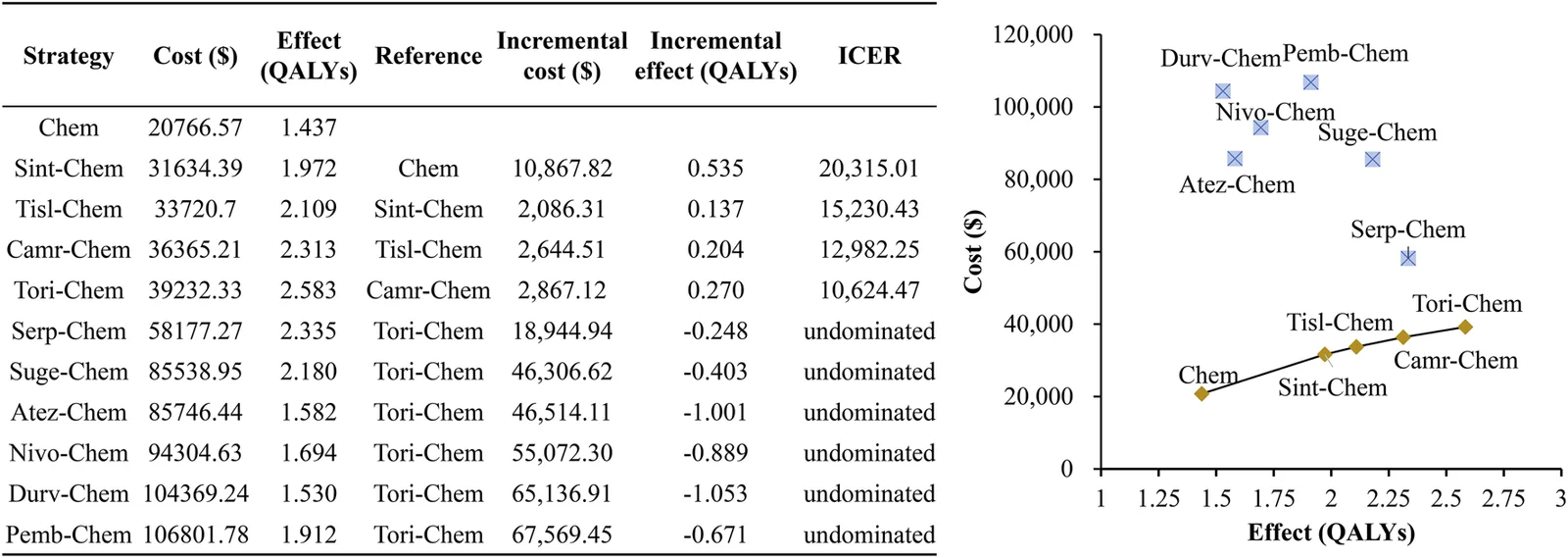
Base-case cost-effectiveness analysis and efficiency frontier of treatment strategies. QALYs, quality-adjusted life-years; ICER, incremental cost-effectiveness ratio; Chem, chemotherapy; Sint-Chem, sintilimab with chemotherapy; Tisl-Chem, tislelizumab with chemotherapy; Camr-Chem, camrelizumab with chemotherapy; Tori-Chem, toripalimab with chemotherapy; Serp-Chem, serplulimab with chemotherapy; Suge-Chem, sugemalimab with chemotherapy; Atez-Chem, atezolizumab with chemotherapy; Nivo-Chem, nivolumab with chemotherapy; Durv-Chem, durvalumab with chemotherapy; Pemb-Chem, pembrolizumab with chemotherapy.

The results of the base-case cost-effectiveness analysis are summarized in Table 2. Chemotherapy alone was associated with the lowest total cost ($20,766.57), whereas Tori-Chem generated the highest QALYs (2.583). Compared with chemotherapy alone, Sint-Chem, Tisl-Chem, and Camr-Chem, Tori-Chem was cost-effective, with ICERs of $16,120.12/QALY, $12,444.52/QALY, $11,638.67/QALY, and $10,624.47/QALY, respectively. Compared with Serp-Chem, Suge-Chem, Atez-Chem, Nivo-Chem, Durv-Chem, and Pemb-Chem, Tori-Chem was an economically dominant strategy, providing greater health benefits at lower total costs. As shown in Figure 3, chemotherapy alone, Sint-Chem, Tisl-Chem, Camr-Chem, and Tori-Chem constituted the efficiency frontier, indicating that these strategies were non-dominated.

**TABLE 2:** Cost-effectiveness of PD-1/PD-L1 inhibitor with chemotherapy in non-squamous NSCLC patients.

Strategy	Cost ($)	Effect (QALYs)	Incremental effect (Cost/QALY)				
			Vs. Chem	Vs. Sint-Chem	Vs. Tisl-Chem	Vs. Carm-Chem	Vs. Tori-Chem
Chem	20,766.57	1.437	-	​	​	​	​
Sint-Chem	31,634.39	1.972	20,315.01	-	​	​	​
Tisl-Chem	33,720.70	2.109	19,278.47	15,230.43	-	​	​
Camr-Chem	36,365.21	2.313	17,813.78	13,886.20	12,982.25	-	​
Tori-Chem	39,232.33	2.583	16,120.12	12,444.52	11,638.67	10,624.47	-
Serp-Chem	58,177.27	2.335	41,694.43	73,263.37	108,545.83	1,009,397.01	undominated
Suge-Chem	85,538.95	2.180	87,203.58	259,397.23	731,648.17	undominated	undominated
Atez-Chem	85,746.44	1.582	449,236.89	undominated	undominated	undominated	undominated
Nivo-Chem	94,304.63	1.694	286,475.83	undominated	undominated	undominated	undominated
Durv-Chem	104,369.24	1.530	905,496.38	undominated	undominated	undominated	undominated
Pemb-Chem	106,801.78	1.912	181,400.58	undominated	undominated	undominated	undominated

QALYs, quality-adjusted life-years; ICER, incremental cost-effectiveness ratio; Chem, chemotherapy; Sint-Chem, sintilimab with chemotherapy; Tisl-Chem, tislelizumab with chemotherapy; Camr-Chem, camrelizumab with chemotherapy; Tori-Chem, toripalimab with chemotherapy; Serp-Chem, serplulimab with chemotherapy; Suge-Chem, sugemalimab with chemotherapy; Atez-Chem, atezolizumab with chemotherapy; Nivo-Chem, nivolumab with chemotherapy; Durv-Chem, durvalumab with chemotherapy; Pemb-Chem, pembrolizumab with chemotherapy.

Using LYs as the effectiveness outcome produced results consistent with the primary QALY-based analysis, with Tori-Chem remaining the most cost-effective strategy (Supplementary Table S11). Similarly, Tori-Chem, Tisl-Chem, Camr-Chem, and Sint-Chem generated the highest NMB in the NMB analysis (Table 3). Under the predefined WTP threshold of $41,859 per QALY, the treatment strategies could be ranked as follows: Tori-Chem > Camr-Chem > Tisl-Chem > Sint-Chem > chemotherapy alone.

**TABLE 3:** Net monetary benefit of PD-1/PD-L1 inhibtors plus chemotherapy in non-squamous NSCLC patients.

Strategy	Cost ($)	QALY			LY		
		Effect (QALYs)	NMB	Rank	Effect (LYs)	NMB	Rank
Chem	20,766.57	1.437	39,403.73	6	2.449	81,757.21	6
Sint-Chem	31,634.39	1.972	50,929.01	4	3.297	106,379.34	4
Tisl-Chem	33,720.70	2.109	54,576.67	3	3.365	107,132.45	3
Camr-Chem	36,365.21	2.313	60,458.93	2	3.713	119,061.07	2
Tori-Chem	39,232.33	2.583	68,887.88	1	4.552	151,296.57	1
Serp-Chem	58,177.27	2.335	39,551.40	5	3.690	96,274.28	5
Suge-Chem	85,538.95	2.180	5,723.05	7	3.719	70,139.07	7
Atez-Chem	85,746.44	1.582	−19,521.44	8	2.563	21,558.02	8
Nivo-Chem	94,304.63	1.694	−23,389.16	9	2.622	15,466.12	9
Durv-Chem	104,369.24	1.530	−40,334.18	11	2.396	−4,081.98	11
Pemb-Chem	106,801.78	1.912	−26,778.46	10	3.036	20,297.21	10

NMB, net monetary benefit, was calculated: QALYs (LYs) × WTP − Cost, with NMB >0 indicating that the regimen provides economic benefit under the current WTP threshold; QALYs, quality-adjusted life-years; WTP, willingness-to-pay ($41,859); ICER, incremental cost-effectiveness ratio; Chem, chemotherapy; Sint-Chem, sintilimab with chemotherapy; Tisl-Chem, tislelizumab with chemotherapy; Camr-Chem, camrelizumab with chemotherapy; Tori-Chem, toripalimab with chemotherapy; Serp-Chem, serplulimab with chemotherapy; Suge-Chem, sugemalimab with chemotherapy; Atez-Chem, atezolizumab with chemotherapy; Nivo-Chem, nivolumab with chemotherapy; Durv-Chem, durvalumab with chemotherapy; Pemb-Chem, pembrolizumab with chemotherapy.

### 3.3 Sensitivity analyses

**FIGURE 4:**
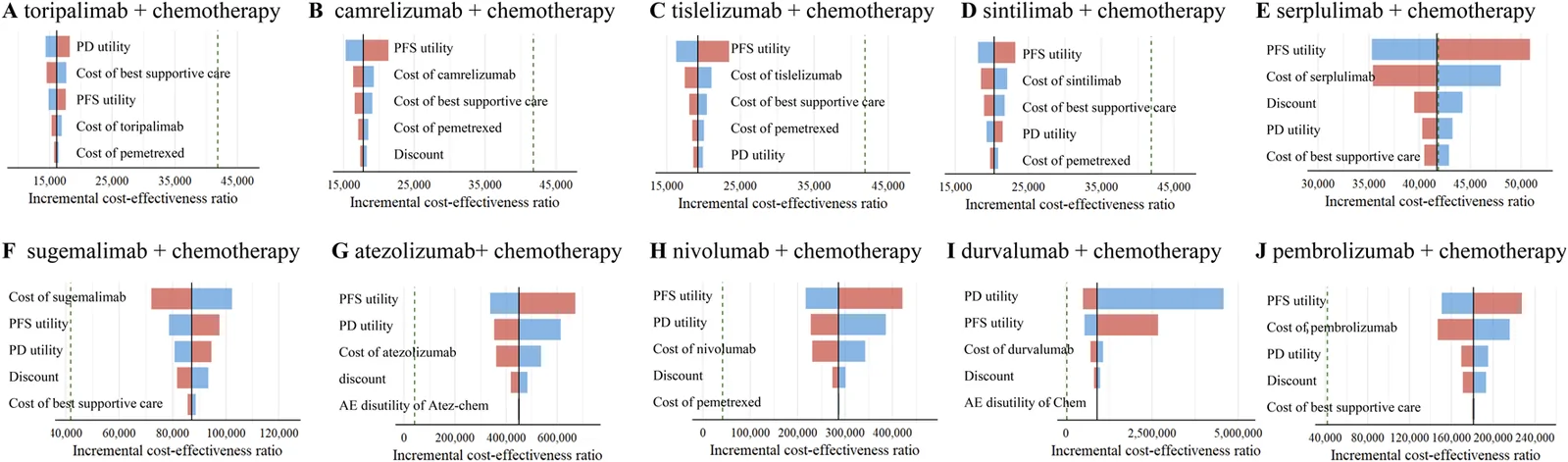
One-way sensitivity analysis of key parameters affecting incremental cost-effectiveness ratio campared with chemotherapy. **(A)** toripalimab with chemotherapy; **(B)** camrelizumab with chemotherapy; **(C)** tislelizumab with chemotherapy; **(D)** sintilimab with chemotherapy; **(E)** serplulimab with chemotherapy; **(F)** sugemalimab with chemotherapy; **(G)** atezolizumab with Chemotherapy; **(H)** nivolumab with chemotherapy; **(I)** durvalumab with chemotherapy; **(J)** pembrolizumab with chemotherapy. PD, progression disease; PFS, progression-free survival; sAEs, grade ≥3 adverse events.

The DSA was performed using the ICER relative to chemotherapy as the outcome measure. The model results were most sensitive to the utility values for the PD and PFS health states and the acquisition costs of PD-1/PD-L1 inhibitors. However, variations in these parameters did not materially alter the base-case conclusions, indicating that the model was robust overall. Serp-Chem represented the only regimen for which cost-effectiveness was sensitive to plausible parameter uncertainty (Figure 4E). The base-case ICER ($41,694.43/QALY) was close to the predefined WTP threshold ($41,859/QALY). Consequently, variations in key model parameters had a substantial impact on its cost-effectiveness.

**FIGURE 5:**
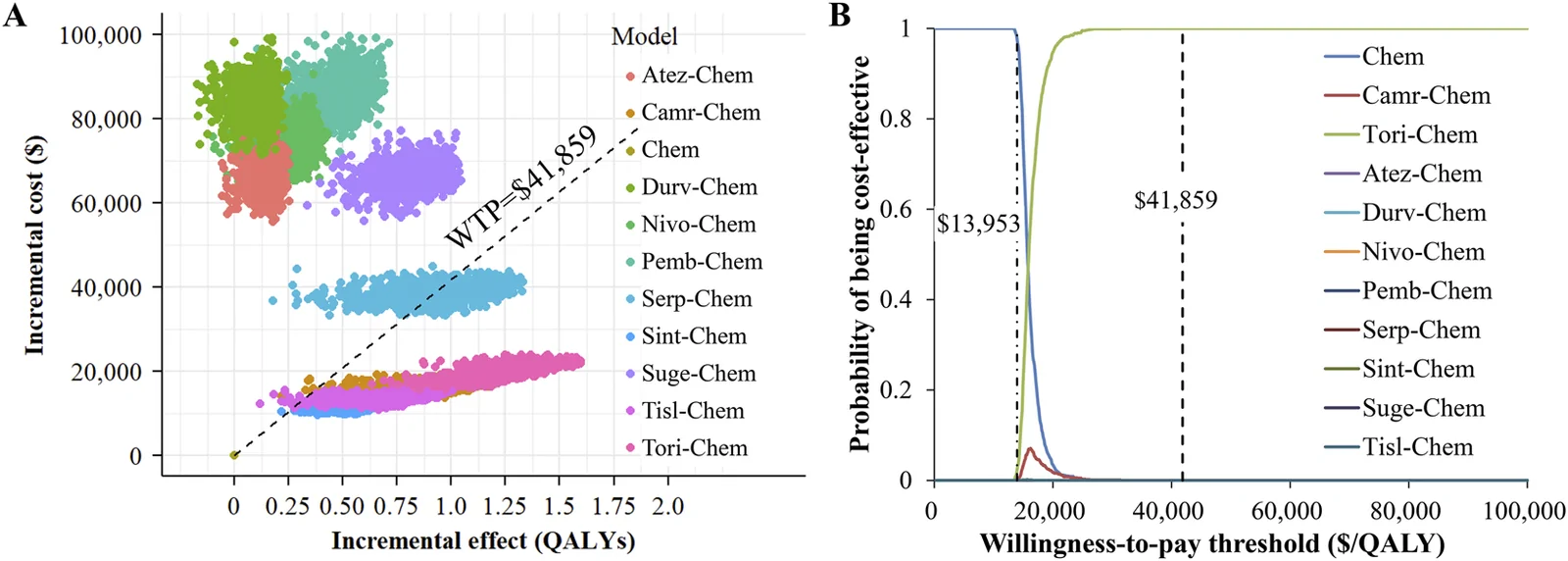
Probabilistic sensitivity analysis. **(A)** scatter plot from Markov model simulations; **(B)** cost-effectiveness acceptability curve. QALYs, quality-adjusted life-years; ICER, incremental cost-effectiveness ratio; Chem, chemotherapy; Sint-Chem, sintilimab with chemotherapy; Tisl-Chem, tislelizumab with chemotherapy; Camr-Chem, camrelizumab with chemotherapy; Tori-Chem, toripalimab with chemotherapy; Serp-Chem, serplulimab with chemotherapy; Suge-Chem, sugemalimab with chemotherapy; Atez-Chem, atezolizumab with chemotherapy; Nivo-Chem, nivolumab with chemotherapy; Durv-Chem, durvalumab with chemotherapy; Pemb-Chem, pembrolizumab with chemotherapy.

PSA demonstrated that, across 1,000 Monte Carlo simulations, compared with chemotherapy alone, the probabilities of being cost-effective at the predefined WTP threshold were 100.0% for Tori-Chem, 99.9% for Sint-Chem, 99.3% for Camr-Chem, 98.5% for Tisl-Chem, and 64.3% for Serp-Chem (Figure 5A). The cost-effectiveness acceptability curve (Figure 5B) showed that, as the WTP threshold increased from $0 to $41,859 (0–3× the 2025 GDP *per capita*), the probability that chemotherapy alone would achieve the highest NMB decreased from 100% to 0%. Conversely, the probability that Tori-Chem would achieve the highest expected NMB increased from 0% to 100%. Even when the WTP threshold was increased to 10 times GDP *per capita*, the remaining treatment strategies retained probabilities close to zero of achieving the highest NMB.

## 4 Discussion

This study provides a comprehensive evaluation of the comparative clinical and economic value of first-line PD-1/PD-L1 inhibitor–based combination therapies for patients with advanced driver mutation–negative non-squamous NSCLC. We achieved this by integrating a NMA with a treatment-specific cost-effectiveness model. Based on indirect comparisons from the NMA, this study demonstrated that Tori-Chem, Sint-Chem, and Pemb-Chem exhibited favorable efficacy and safety profiles among the 13 PD-1/PD-L1 inhibitor-based combination regimens evaluated. Furthermore, the cost-effectiveness analysis, conducted from the perspective of the Chinese healthcare system, indicated that Tori-Chem provided favorable cost-effectiveness while maintaining superior clinical outcomes. These findings suggest that treatment selection should incorporate not only clinical efficacy and safety but also economic value to maximize patient benefit and optimize healthcare resource allocation.

The NMA approach allowed indirect comparisons among different PD-1/PD-L1 inhibitor-based regimens, providing robust evidence for efficacy and safety in the absence of head-to-head trials. Consistent with previous studies, our results further support that PD-1/PD-L1 inhibitor-based combination therapy significantly improved PFS and OS while increasing ORR compared with chemotherapy alone, and have increasingly become a recommended first-line treatment (Rao et al., 2025; Xu et al., 2025; Liu et al., 2025). These combination regimens enhance the immune system’s ability to recognize and eliminate tumor cells, resulting in substantial survival benefits (Li et al., 2024). However, improved efficacy was accompanied by an increased incidence of AEs and sAEs. In particular, Camr-Chem, Sint-Chem, and Nivo-Chem were associated with relatively higher risks of sAEs, whereas Tori-Chem demonstrated a comparatively favorable safety profile, with a sAEs risk similar to that of chemotherapy alone. Although the biological mechanisms underlying these differences remain not fully understood, variations in antibody characteristics, patient populations, chemotherapy backbones, and treatment exposure may contribute to the observed differences in toxicity profiles. These findings highlight the importance of balancing treatment efficacy with tolerability, particularly in patients at increased risk of treatment-related toxicities.

From an economic perspective, we developed a Markov model simulating 10-year outcomes for 11 regimens based on the Chinese healthcare system. Tori-Chem demonstrated the highest overall economic value among all evaluated strategies. This finding reflects the favorable balance between incremental health benefits and additional treatment costs. Compared with other PD-1/PD-L1 inhibitor–based regimens, Tori-Chem achieved the greatest QALY gain while maintaining relatively low drug acquisition costs and a lower incidence of sAEs, resulting in both favorable ICERs and the highest NMB under the predefined WTP threshold. These findings are consistent with previous economic evaluations reporting an ICER of approximately $9,445/QALY for Tori-Chem versus chemotherapy alone, supporting its favorable cost-effectiveness (Zhang et al., 2023). Similarly, Sint-Chem demonstrated an ICER of CNY 220,963.22/QALY relative to chemotherapy in the ORIENT-11 study, which fell within China’s WTP threshold of CNY 242,928/QALY, further confirming its economic value (Huang et al., 2025). Notably, to our knowledge, this study is the first to evaluate the cost-effectiveness of Serp-Chem for patients with non-squamous NSCLC using the final survival analysis data from the pivotal clinical trial (Hao et al., 2026). Compared with chemotherapy alone, Serp-Chem yielded an ICER of $41,694.43/QALY, which was slightly below the predefined WTP threshold ($41,859/QALY), indicating that it was likely to be cost-effective under the current pricing scenario. DSA showed that variations in utility values and drug costs could alter its cost-effectiveness because the base-case ICER was close to the WTP threshold. Nevertheless, probabilistic sensitivity analysis demonstrated that Serp-Chem remained cost-effective in 64.3% of simulations, suggesting moderate robustness of the economic findings.

Across all evaluated regimens, DSA consistently identified health utility values and drug acquisition costs as the major drivers of economic outcomes. PSA further indicated that the cost-effectiveness of the preferred treatment strategies remained relatively stable across WTP thresholds ranging from 3 to 10 times GDP *per capita*, reflecting the overall robustness of the model. Nevertheless, improving the affordability and accessibility of PD-1/PD-L1 inhibitors remains essential for maximizing their clinical and economic value. Previous studies have shown that price reductions and patient assistance programs can substantially improve the cost-effectiveness of pembrolizumab (Jiang and Wang, 2022; Wan et al., 2025). In China, national initiatives such as centralized drug procurement and insurance reimbursement have significantly alleviated patients’ financial burden. Our findings offer robust scientific evidence to inform policy decisions on drug pricing and reimbursement, thereby supporting rational resource allocation and enhancing patient access to therapies (Wang et al., 2024; Yip et al., 2012). Collectively, our findings would support drug pricing negotiations, reimbursement decisions, and efficient allocation of healthcare resources, while also informing evidence-based treatment selection in clinical practice.

Compared with previous studies, the present analysis provides several important methodological and clinical advances. First, our network meta-analysis incorporated the most up-to-date evidence, including recently published phase III trials and updated long-term survival results, thereby providing a more comprehensive comparison of currently available PD-1/PD-L1 inhibitor–based regimens. Second, rather than relying on a single reference survival curve with hazard ratio adjustments, we reconstructed treatment-specific individual patient data from published Kaplan–Meier curves for each regimen included in the economic evaluation and fitted independent parametric survival models. This approach better captures differences in survival patterns among PD-1/PD-L1 inhibitors, particularly the delayed treatment effects and heterogeneous long-term survival associated with immunotherapy, thereby reducing uncertainty in long-term survival extrapolation. Third, to our knowledge, this is the first study to incorporate the mature overall survival results of serplulimab into a cost-effectiveness analysis for patients with advanced non-squamous NSCLC, providing new evidence regarding the economic value of this emerging therapeutic option in China.

Several limitations should be acknowledged. First, the NMA lacked closed loops, and only Part 2 of the CheckMate 227 trial was included, limiting consistency assessment and potentially introducing uncertainty into the comparative estimates. Second, due to differences in regulatory approval status and market availability among PD-1/PD-L1 inhibitors, not all strategies could be evaluated comprehensively for cost-effectiveness; nevertheless, the analysis included the most commonly used regimens in Chinese clinical practice, ensuring practical applicability. Third, although reconstructed IPD and treatment-specific survival modeling were applied to reduce uncertainty associated with survival extrapolation, the absence of original individual-level trial data may still introduce residual uncertainty in long-term survival estimates. Finally, as most regimens analyzed are currently available only in China, the economic evaluation primarily reflects the Chinese healthcare system, and the generalizability to other countries or regions warrants further investigation.

## 5 Conclusion

In conclusion, among currently available first-line treatment strategies for patients with advanced driver mutation–negative non-squamous NSCLC, Tori-Chem, Sint-Chem, and Pemb-Chem demonstrated favorable efficacy and safety, with Tori-Chem showing the greatest overall economic value. These findings may support evidence-based treatment selection and healthcare resource allocation in China. Further multicenter studies with longer follow-up are warranted to validate their long-term clinical outcomes and economic value in real-world clinical practice.

## Data Availability

The original contributions presented in the study are included in the article/Supplementary Material, further inquiries can be directed to the corresponding author.
