# Supplementary material for: Optimizing PD-1/PD-L1 inhibitors plus chemotherapy for driver mutation–negative non-squamous NSCLC in China: a cost-effectiveness analysis
Source: Front Pharmacol. 2026 Aug 21;17:1849145. doi: 10.3389/fphar.2026.1849145 (PMC13541720; doi:10.3389/fphar.2026.1849145)
Supplement: Supplementary file 1 [file Table1.docx]

Supplementary Material

**Supplementary Figures**

**Figure S1** Kaplan-Meier curve of overall survival according to reconstructed individual patient data

**Figure S2** Kaplan-Meier curve of progression-free survival according to reconstructed individual patient data

**Figure S3** Plot for alternate parametric overall survival distributions

**Figure S4** Plot for alternate parametric overall progression-free survival distributions

**Figure S5** Overview of the study selection process

**Figure S6** The efficacy and safety in Network Meta-analysis. (A) objective response rate; (B) adverse events; (C) treatment discontinuation due to adverse events.

**Supplementary tables**

**Table S1** PICOS framework and eligibility criteria of the systematic review

**Table S2** The details of search in PubMed

**Table S3** The heterogeneity and publication bias analysis of all meta-analyses

**Table S4** The CHEERS 2022 checklist

**Table S5** Summary of Statistical Goodness-of-fit of Kaplan-Meier Curve

**Table S6** Summary of parametric equations for progression-free survival and overall survival

**Table S7** Treatment strategies compared in the model

**Table S8** The cost and disutility of grade ≥3 adverse events

**Table S9** The details of including trails

**Table S10** The score of the surface under the cumulative ranking in effectiveness and safety

**Table S11** Cost-effectiveness of PD-1/PD-L1 inhibitor with chemotherapy in non-squamous NSCLC patients base on life-year

# 1 Supplementary Figures and Tables

## 1.1 Supplementary Figures

**Figure S1 Kaplan-Meier curve of overall survival according to reconstructed individual patient data**

**Figure S2 Kaplan-Meier curve of progression-free survival according to reconstructed individual patient data**

**Figure S3 Plot for alternate parametric overall survival distributions**

**Figure S4 Plot for alternate parametric overall progression-free survival distributions**

**Figure S5 Overview of the study selection process**

**Figure S6 The efficacy and safety in Network Meta-analysis. (A) objective response rate; (B) adverse events; (C) treatment discontinuation due to adverse events.**

## 1.2 Supplementary tables

**Table S1 PICOS framework and eligibility criteria of the systematic review**

| PICOS | Criteria |
| --- | --- |
| population | non-squamous non-small cell lung cancer without EGFR sensitizing mutations or ALK rearrangements, without specific PD-L1 expression levels or tumor proportion score (TPS) categories |
| intervention | PD-1/PD-L1 inhibitors combined with platinum-based chemotherapy, without bevacizumab or other anti-angiogenic agents, as frist line treatment |
| comparison | platinum-based chemotherapy, without bevacizumab or other anti-angiogenic agents, as first line treatment |
| outcome | Overall survival, Progression-free survival |
| study design | phase III randomized controlled trials |

**Table S2 The details of search in PubMed**

| **Search number** | **Query** | **Results** | **Date** |
| --- | --- | --- | --- |
| #1 | 'Carcinoma, Non-Small-Cell Lung'[Title/Abstract] OR 'Carcinoma, Non Small Cell Lung'[Title/Abstract] OR 'Carcinomas, Non-Small-Cell Lung'[Title/Abstract] OR 'Lung Carcinoma, Non-Small-Cell'[Title/Abstract] OR 'Lung Carcinomas, Non-Small-Cell'[Title/Abstract] OR 'Non-Small-Cell Lung Carcinomas'[Title/Abstract] OR 'Carcinoma, Non-Small Cell Lung'[Title/Abstract] OR 'Non-Small Cell Lung Cancer'[Title/Abstract] OR 'Non-Small-Cell Lung Carcinoma'[Title/Abstract] OR 'Non Small Cell Lung Carcinoma'[Title/Abstract] OR 'Nonsmall Cell Lung Cancer'[Title/Abstract] OR 'Non-Small Cell Lung Carcinoma'[Title/Abstract] OR 'NSCLC'[Title/Abstract] | 108,427 | 2025/12/10 |
| #2 | ('non squamous'[Title/Abstract] OR 'non-squamous'[Title/Abstract]) OR (nonsquamous[Title/Abstract]) | 3,366 | 2025/12/10 |
| #3 | #1 AND #2 | 2,543 | 2025/12/10 |
| #4 | 'Immune Checkpoint Inhibitor'[Title/Abstract] OR 'Checkpoint Inhibitors, Immune'[Title/Abstract] OR 'Immune Checkpoint Blockers'[Title/Abstract] OR 'Checkpoint Blockers, Immune'[Title/Abstract] OR 'Immune Checkpoint Inhibitor'[Title/Abstract] OR 'Checkpoint Inhibitor, Immune'[Title/Abstract] OR 'PD-1 Inhibitors'[Title/Abstract] OR 'PD 1 Inhibitors'[Title/Abstract] OR 'Programmed Cell Death Protein 1 Inhibitor'[Title/Abstract] OR 'Programmed Cell Death Protein 1 Inhibitors'[Title/Abstract] OR 'PD-1 Inhibitor'[Title/Abstract] OR 'Inhibitor, PD-1'[Title/Abstract] OR 'PD 1 Inhibitor'[Title/Abstract] OR 'Immune Checkpoint Blockade'[Title/Abstract] OR 'Checkpoint Blockade, Immune'[Title/Abstract] OR 'Immune Checkpoint Inhibition'[Title/Abstract] OR 'Checkpoint Inhibition, Immune'[Title/Abstract] OR 'PD-L1 Inhibitors'[Title/Abstract] OR 'PD L1 Inhibitors'[Title/Abstract] OR 'Programmed Death-Ligand 1 Inhibitors'[Title/Abstract] OR 'Programmed Death Ligand 1 Inhibitors'[Title/Abstract] OR 'PD-L1 Inhibitor'[Title/Abstract] OR 'PD L1 Inhibitor'[Title/Abstract] OR 'PD-1-PD-L1 Blockade'[Title/Abstract] OR 'Blockade, PD-1-PD-L1'[Title/Abstract] OR 'PD 1 PD L1 Blockade'[Title/Abstract] | 31,151 | 2025/12/10 |
| #5 | 'pembrolizumab'[Title/Abstract] OR 'MK-3475'[Title/Abstract] OR 'Keytruda'[Title/Abstract] OR 'lambrolizumab'[Title/Abstract] OR 'SCH-900475'[Title/Abstract] OR 'nivolumab'[Title/Abstract] OR 'MDX-1106'[Title/Abstract] OR 'MDX1106'[Title/Abstract] OR 'MDX 1106'[Title/Abstract] OR 'Opdivo'[Title/Abstract] OR 'BMS-936558'[Title/Abstract] OR 'BMS936558'[Title/Abstract] OR 'BMS 936558'[Title/Abstract] OR 'ONO-4538'[Title/Abstract] OR 'ONO4538'[Title/Abstract] OR 'ONO 4538'[Title/Abstract] OR 'sintilimab '[Title/Abstract] OR 'IBI 308'[Title/Abstract] OR 'IBI308'[Title/Abstract] OR 'IBI-308'[Title/Abstract] OR 'atezolizumab'[Title/Abstract] OR 'anti-PDL1'[Title/Abstract] OR 'MPDL3280A'[Title/Abstract] OR 'MPDL-3280A'[Title/Abstract] OR 'Tecentriq'[Title/Abstract] OR 'RG7446'[Title/Abstract] OR 'RG-7446'[Title/Abstract] OR 'camrelizumab'[Title/Abstract] OR 'SHR-1210'[Title/Abstract] OR 'SHR 1210'[Title/Abstract] OR 'carrelizumab'[Title/Abstract] OR 'durvalumab'[Title/Abstract] OR 'MEDI4736'[Title/Abstract] OR 'MEDI-4736'[Title/Abstract] OR 'Imfinzi'[Title/Abstract] OR 'tislelizumab '[Title/Abstract] OR 'tislelizumab-jsgr'[Title/Abstract] OR 'BGB-A317'[Title/Abstract] OR 'JHL-2108'[Title/Abstract] OR 'JHL2108'[Title/Abstract] OR 'tevimbra'[Title/Abstract] OR 'toripalimab '[Title/Abstract] OR 'toripalimab-tpzi'[Title/Abstract] OR 'sugemalimab '[Title/Abstract] OR 'Serplulimab'[Title/Abstract] | 28,117 | 2025/12/10 |
| #6 | #4 OR #5 | 51,400 | 2025/12/8 |
| #7 | 'Clinical Trials, Randomized'[Title/Abstract] OR 'Trials, Randomized Clinical'[Title/Abstract] OR 'Controlled Clinical Trials, Randomized'[Title/Abstract] OR 'randomized clinical trial '[Title/Abstract] OR 'RCT'[Title/Abstract] OR 'clinical trial*'[Title/Abstract] OR ' trial*'[Title/Abstract] OR ' phase III'[Title/Abstract] OR 'phase 3'[Title/Abstract] OR 'clinical trial'[Title/Abstract] OR 'Randomized Controlled Trials as Topic'[Title/Abstract] | 1,609,229 | 2025/12/10 |
| #8 | #3 AND #6 AND #7 | 361 | 2025/12/10 |
|  |  |  |  |
| **Search number** | **Query** | **Results** | **Update** |
| 1 | 'Carcinoma, Non-Small-Cell Lung'[Title/Abstract] OR 'Carcinoma, Non Small Cell Lung'[Title/Abstract] OR 'Carcinomas, Non-Small-Cell Lung'[Title/Abstract] OR 'Lung Carcinoma, Non-Small-Cell'[Title/Abstract] OR 'Lung Carcinomas, Non-Small-Cell'[Title/Abstract] OR 'Non-Small-Cell Lung Carcinomas'[Title/Abstract] OR 'Carcinoma, Non-Small Cell Lung'[Title/Abstract] OR 'Non-Small Cell Lung Cancer'[Title/Abstract] OR 'Non-Small-Cell Lung Carcinoma'[Title/Abstract] OR 'Non Small Cell Lung Carcinoma'[Title/Abstract] OR 'Nonsmall Cell Lung Cancer'[Title/Abstract] OR 'Non-Small Cell Lung Carcinoma'[Title/Abstract] OR 'NSCLC'[Title/Abstract] | 113,580 | 2026/7/1 |
| 2 | ('non squamous'[Title/Abstract] OR 'non-squamous'[Title/Abstract]) OR (nonsquamous[Title/Abstract]) | 3,515 | 2026/7/1 |
| 4 | 'Immune Checkpoint Inhibitor'[Title/Abstract] OR 'Checkpoint Inhibitors, Immune'[Title/Abstract] OR 'Immune Checkpoint Blockers'[Title/Abstract] OR 'Checkpoint Blockers, Immune'[Title/Abstract] OR 'Immune Checkpoint Inhibitor'[Title/Abstract] OR 'Checkpoint Inhibitor, Immune'[Title/Abstract] OR 'PD-1 Inhibitors'[Title/Abstract] OR 'PD 1 Inhibitors'[Title/Abstract] OR 'Programmed Cell Death Protein 1 Inhibitor'[Title/Abstract] OR 'Programmed Cell Death Protein 1 Inhibitors'[Title/Abstract] OR 'PD-1 Inhibitor'[Title/Abstract] OR 'Inhibitor, PD-1'[Title/Abstract] OR 'PD 1 Inhibitor'[Title/Abstract] OR 'Immune Checkpoint Blockade'[Title/Abstract] OR 'Checkpoint Blockade, Immune'[Title/Abstract] OR 'Immune Checkpoint Inhibition'[Title/Abstract] OR 'Checkpoint Inhibition, Immune'[Title/Abstract] OR 'PD-L1 Inhibitors'[Title/Abstract] OR 'PD L1 Inhibitors'[Title/Abstract] OR 'Programmed Death-Ligand 1 Inhibitors'[Title/Abstract] OR 'Programmed Death Ligand 1 Inhibitors'[Title/Abstract] OR 'PD-L1 Inhibitor'[Title/Abstract] OR 'PD L1 Inhibitor'[Title/Abstract] OR 'PD-1-PD-L1 Blockade'[Title/Abstract] OR 'Blockade, PD-1-PD-L1'[Title/Abstract] OR 'PD 1 PD L1 Blockade'[Title/Abstract] | 35,190 | 2026/7/1 |
| 5 | (('pembrolizumab'[Title/Abstract] OR 'MK-3475'[Title/Abstract] OR 'Keytruda'[Title/Abstract] OR 'lambrolizumab'[Title/Abstract] OR 'SCH-900475'[Title/Abstract] OR 'nivolumab'[Title/Abstract] OR 'MDX-1106'[Title/Abstract] OR 'MDX1106'[Title/Abstract] OR 'MDX 1106'[Title/Abstract] OR 'Opdivo'[Title/Abstract] OR 'BMS-936558'[Title/Abstract] OR 'BMS936558'[Title/Abstract] OR 'BMS 936558'[Title/Abstract] OR 'ONO-4538'[Title/Abstract] OR 'ONO4538'[Title/Abstract] OR 'ONO 4538'[Title/Abstract] OR 'sintilimab '[Title/Abstract] OR 'IBI 308'[Title/Abstract] OR 'IBI308'[Title/Abstract] OR 'IBI-308'[Title/Abstract] OR 'atezolizumab'[Title/Abstract] OR 'anti-PDL1'[Title/Abstract] OR 'MPDL3280A'[Title/Abstract] OR 'MPDL-3280A'[Title/Abstract] OR 'Tecentriq'[Title/Abstract] OR 'RG7446'[Title/Abstract] OR 'RG-7446'[Title/Abstract] OR 'camrelizumab'[Title/Abstract] OR 'SHR-1210'[Title/Abstract] OR 'SHR 1210'[Title/Abstract] OR 'carrelizumab'[Title/Abstract] OR 'durvalumab'[Title/Abstract] OR 'MEDI4736'[Title/Abstract] OR 'MEDI-4736'[Title/Abstract] OR 'Imfinzi'[Title/Abstract] OR 'tislelizumab '[Title/Abstract] OR 'tislelizumab-jsgr'[Title/Abstract] OR 'BGB-A317'[Title/Abstract] OR 'JHL-2108'[Title/Abstract] OR 'JHL2108'[Title/Abstract] OR 'tevimbra'[Title/Abstract] OR 'toripalimab '[Title/Abstract] OR 'toripalimab-tpzi'[Title/Abstract] OR 'sugemalimab '[Title/Abstract] OR 'Serplulimab'[Title/Abstract]) OR (Prolgolimab[Title/Abstract])) OR (BCD-100[Title/Abstract]) | 31,311 | 2026/7/1 |
| 6 | #4 OR #5 | 57,706 | 2026/7/1 |
| 7 | #1 AND #2 | 2,674 | 2026/7/1 |
| 8 | 'Clinical Trials, Randomized'[Title/Abstract] OR 'Trials, Randomized Clinical'[Title/Abstract] OR 'Controlled Clinical Trials, Randomized'[Title/Abstract] OR 'randomized clinical trial '[Title/Abstract] OR 'RCT'[Title/Abstract] OR 'clinical trial*'[Title/Abstract] OR ' trial*'[Title/Abstract] OR ' phase III'[Title/Abstract] OR 'phase 3'[Title/Abstract] OR 'clinical trial'[Title/Abstract] OR 'Randomized Controlled Trials as Topic'[Title/Abstract] | 1,691,658 | 2026/7/1 |
| 9 | #6 AND #7 AND #8 | 395 | 2026/7/1 |

**Table S3 The heterogeneity and publication bias analysis of all meta-analyses in the present study**

| Network meta-analysis | Heterogeneity | | Publication bias |
| --- | --- | --- | --- |
|  | P value for Q test | *I*^2^ | Egger's test |
| overall survival | 0.5694 | 0% | 0.8431 |
| progression-free survival | 0.6289 | 0% | 0.7829 |
| objective response rate | 0.7106 | 0% | 0.9721 |
| adverse events | 0.5266 | 0% | 0.7907 |
| grade≥3 adverse events | 0.891 | 0% | 0.9842 |
| treatment discontinuation due to adverse events | 0.224 | 32% | 0.845 |

**Table S4 The CHEERS 2022 checklist**

| **Topic** | **No.** | **Item** | **Location where item is reported** |
| --- | --- | --- | --- |
| **Title** |  |  |  |
|  | 1 | Identify the study as an economic evaluation and specify the interventions being compared. | Title, Page 1 |
| **Abstract** |  |  |  |
|  | 2 | Provide a structured summary that highlights context, key methods, results, and alternative analyses. | Abstract, Page 1 |
| **Introduction** |  |  |  |
| **Background and objectives** | 3 | Give the context for the study, the study question, and its practical relevance for decision making in policy or practice. | Introduction Paragraph 1-3 |
| **Methods** |  |  |  |
| **Health economic analysis plan** | 4 | Indicate whether a health economic analysis plan was developed and where available. | Not applicable |
| **Study population** | 5 | Describe characteristics of the study population (such as age range, demographics, socioeconomic, or clinical characteristics). | Methods Section 2.3 |
| **Setting and location** | 6 | Provide relevant contextual information that may influence findings. | Methods Section 2.5 Paragraph 1 |
| **Comparators** | 7 | Describe the interventions or strategies being compared and why chosen. | Methods Section 2.5 Paragraph 2 |
| **Perspective** | 8 | State the perspective(s) adopted by the study and why chosen. | Methods Section 2.5 Paragraph 1 |
| **Time horizon** | 9 | State the time horizon for the study and why appropriate. | Methods Section 2.3 |
| **Discount rate** | 10 | Report the discount rate(s) and reason chosen. | Methods Section 2.5 Paragraph 1 |
| **Selection of outcomes** | 11 | Describe what outcomes were used as the measure(s) of benefit(s) and harm(s). | Methods Section 2.5 Paragraph 2 |
| **Measurement of outcomes** | 12 | Describe how outcomes used to capture benefit(s) and harm(s) were measured. | Methods Section 2.5 Paragraph 2 |
| **Valuation of outcomes** | 13 | Describe the population and methods used to measure and value outcomes. | Methods Section 2.5 Paragraph 2 |
| **Measurement and valuation of resources and costs** | 14 | Describe how costs were valued. | Not reported |
| **Currency, price date, and conversion** | 15 | Report the dates of the estimated resource quantities and unit costs, plus the currency and year of conversion. | Methods Section 2.5 Paragraph 1 |
| **Rationale and description of model** | 16 | If modelling is used, describe in detail and why used. Report if the model is publicly available and where it can be accessed. | Methods  Section 2.3 |
| **Analytics and assumptions** | 17 | Describe any methods for analysing or statistically transforming data, any extrapolation methods, and approaches for validating any model used. | Methods Section 2.4 and Appendix |
| **Characterising heterogeneity** | 18 | Describe any methods used for estimating how the results of the study vary for subgroups. | Not applicable |
| **Characterising distributional effects** | 19 | Describe how impacts are distributed across different individuals or adjustments made to reflect priority populations. | Methods Section 2.6 |
| **Characterising uncertainty** | 20 | Describe methods to characterise any sources of uncertainty in the analysis. | Methods Section 2.6 |
| **Approach to engagement with patients and others affected by the study** | 21 | Describe any approaches to engage patients or service recipients, the general public, communities, or stakeholders (such as clinicians or payers) in the design of the study. | Appendix |
| **Results** |  |  |  |
| **Study parameters** | 22 | Report all analytic inputs (such as values, ranges, references) including uncertainty or distributional assumptions. | Results Section 3.2 and Table1 |
| **Summary of main results** | 23 | Report the mean values for the main categories of costs and outcomes of interest and summarise them in the most appropriate overall measure. | Results Section 3.2 |
| **Effect of uncertainty** | 24 | Describe how uncertainty about analytic judgments, inputs, or projections affect findings. Report the effect of choice of discount rate and time horizon, if applicable. | Results Section 3.3-3.4 |
| **Effect of engagement with patients and others affected by the study** | 25 | Report on any difference patient/service recipient, general public, community, or stakeholder involvement made to the approach or findings of the study | Not reported |
| **Discussion** |  |  |  |
| **Study findings, limitations, generalisability, and current knowledge** | 26 | Report key findings, limitations, ethical or equity considerations not captured, and how these could affect patients, policy, or practice. | Discussion |
| **Other relevant information** |  |  |  |
| **Source of funding** | 27 | Describe how the study was funded and any role of the funder in the identification, design, conduct, and reporting of the analysis | No |
| **Conflicts of interest** | 28 | Report authors conflicts of interest according to journal or International Committee of Medical Journal Editors requirements. | Section **Conflicts of interest** |

*From:* Husereau D, Drummond M, Augustovski F, et al. Consolidated Health Economic Evaluation Reporting Standards 2022 (CHEERS 2022) Explanation and Elaboration: A Report of the ISPOR CHEERS II Good Practices Task Force. Value Health 2022;25. <doi:10.1016/j.jval.2021.10.008>

**Table S5 Summary of Statistical Goodness-of-fit of Kaplan-Meier Curve**

| Strategy | Goodness of fit | | Exponential | Weibull | Log-normal | Log-logistic | Gamma | Gompertz |
| --- | --- | --- | --- | --- | --- | --- | --- | --- |
| Camr-Chem |  |  |  |  |  |  |  |  |
|  | PFS | AIC | 1237.2 | 1236.6 | **1204.3** | 1206.4 | 1238.9 | 1217.7 |
|  |  | BIC | 1240.5 | 1243.2 | **1211.0** | 1213.1 | 1245.6 | 1224.3 |
|  | OS | AIC | 1346.4 | 1348.2 | **1328.2** | 1333.3 | 1347.1 | 1345.6 |
|  |  | BIC | 1349.8 | 1354.8 | **1334.8** | 1339.9 | 1353.7 | 1352.3 |
| Atez-Chem |  |  |  |  |  |  |  |  |
|  | PFS | AIC | 3778.6 | 3762.4 | 3742.4 | **3725.8** | 3753.5 | 3780.1 |
|  |  | BIC | 3783.3 | 3771.6 | 3751.6 | **3735.0** | 3762.7 | 3789.3 |
|  | OS | AIC | 3573.7 | 3572.3 | 3563.7 | **3560.2** | 3570.3 | 3575.7 |
|  |  | BIC | 3578.3 | 3581.5 | 3572.9 | **3569.4** | 3579.5 | 3584.9 |
| Pemb-Chem |  |  |  |  |  |  |  |  |
|  | PFS | AIC | 2850.0 | 2845.1 | 2802.3 | **2798.0** | 2850.5 | 2817.9 |
|  |  | BIC | 2854.0 | 2853.1 | 2810.4 | **2806.0** | 2858.5 | 2826.0 |
|  | OS | AIC | 2971.4 | 2973.2 | 2966.3 | **2956.3** | 2973.3 | 2966.3 |
|  |  | BIC | 2975.4 | 2981.3 | 2974.3 | **2964.3** | 2981.4 | 2974.3 |
| Suge-Chem |  |  |  |  |  |  |  |  |
|  | PFS | AIC | 930.5 | 928.0 | **918.1** | 920.1 | 925.9 | 932.4 |
|  |  | BIC | 933.8 | 934.5 | **924.6** | 926.6 | 932.4 | 938.9 |
|  | OS | AIC | 1062.6 | 1064.5 | **1055.7** | 1058.1 | 1064.3 | 1062.7 |
|  |  | BIC | 1065.8 | 1071.0 | **1062.2** | 1064.6 | 1070.8 | 1069.3 |
| Durv-Chem |  |  |  |  |  |  |  |  |
|  | PFS | AIC | 821.2 | 821.6 | 812.6 | **811.8** | 820.1 | 822.6 |
|  |  | BIC | 824.5 | 828.3 | 819.3 | **818.5** | 826.8 | 829.3 |
|  | OS | AIC | 1475.2 | 1470.1 | 1456.1 | **1453.6** | 1473.5 | 1455.8 |
|  |  | BIC | 1478.6 | 1476.8 | 1462.8 | **1460.3** | 1480.2 | 1462.5 |
| Nivo-Chem |  |  |  |  |  |  |  |  |
|  | PFS | AIC | 1356.4 | 1355.1 | 1342.0 | **1341.5** | 1352.6 | 1358.3 |
|  |  | BIC | 1360.0 | 1362.3 | 1349.2 | **1348.7** | 1359.8 | 1365.5 |
|  | OS | AIC | 1341.9 | 1336.0 | 1338.4 | **1333.9** | 1335.1 | 1340.5 |
|  |  | BIC | 1345.5 | 1343.2 | 1345.6 | **1341.1** | 1342.3 | 1347.7 |
| Tisl-Chem |  |  |  |  |  |  |  |  |
|  | PFS | AIC | 1167.6 | 1169.2 | **1139.3** | 1140.6 | 1169.4 | 1155.3 |
|  |  | BIC | 1171.0 | 1176.0 | **1146.1** | 1147.4 | 1176.2 | 1162.1 |
|  | OS | AIC | 1344.7 | 1343.4 | **1315.1** | 1321.8 | 1339.6 | 1345.0 |
|  |  | BIC | 1348.1 | 1350.2 | **1321.9** | 1328.6 | 1346.4 | 1351.8 |
| Sint-Chem |  |  |  |  |  |  |  |  |
|  | PFS | AIC | 817.8 | 802.6 | **799.2** | 801.2 | 801.2 | 810.3 |
|  |  | BIC | 821.3 | 809.8 | **806.4** | 808.4 | 808.4 | 817.5 |
|  | OS | AIC | 1371.0 | 1367.5 | **1358.3** | 1360.3 | 1365.5 | 1372.5 |
|  |  | BIC | 1374.6 | 1374.6 | **1365.5** | 1367.4 | 1372.7 | 1379.7 |
| Tori-Chem |  |  |  |  |  |  |  |  |
|  | PFS | AIC | 2205.7 | 2207.7 | **2146.3** | 2152.3 | 2205.1 | 2185.4 |
|  |  | BIC | 2209.6 | 2215.6 | **2154.2** | 2160.2 | 2213.0 | 2193.3 |
|  | OS | AIC | 908.2 | 910.2 | **899.7** | 905.1 | 910.1 | 905.2 |
|  |  | BIC | 911.7 | 917.1 | **906.7** | 912.0 | 917.0 | 912.1 |
| Serp-Chem |  |  |  |  |  |  |  |  |
|  | PFS | AIC | 1151.4 | 1149.0 | **1118.6** | 1121.2 | 1152.2 | 1127.3 |
|  |  | BIC | 1154.7 | 1155.7 | **1125.3** | 1128.0 | 1158.9 | 1134.1 |
|  | OS | AIC | 1262.6 | 1263.7 | **1246.2** | 1251.2 | 1262.2 | 1263.1 |
|  |  | BIC | 1266.0 | 1270.4 | **1252.9** | 1257.9 | 1268.9 | 1269.8 |
| Chem |  |  |  |  |  |  |  |  |
|  | PFS | AIC | 14437.1 | 14308.8 | **14079.9** | 14094.1 | 14228.5 | 14436.8 |
|  |  | BIC | 14443.0 | 14320.6 | **14091.7** | 14105.9 | 14240.3 | 14448.7 |
|  | OS | AIC | 13161.5 | 13160.3 | **13015.8** | 13030.0 | 13148.8 | 13142.3 |
|  |  | BIC | 13167.2 | 13171.7 | **13027.2** | 13041.3 | 13160.2 | 13153.6 |

**Table S6 Summary of parametric equations for progression-free survival and overall survival**

| Strategy | Survival | Value | Distribution |
| --- | --- | --- | --- |
| Camr-Chem |  |  |  |
|  | PFS | mu = 2.81384, sigma =1.37835 | Log-normal |
|  | OS | mu = 3.72204, sigma =1.3022 | Log-normal |
| Atez-Chem |  |  |  |
|  | PFS | scale = 10.77077, shape =1.57504 | Log-logistic |
|  | OS | scale = 24.45938, shape =1.32979 | Log-logistic |
| Pemb-Chem |  |  |  |
|  | PFS | scale = 13.61072, shape =1.38009 | Log-logistic |
|  | OS | scale = 31.23052, shape =1.40328 | Log-logistic |
| Durv-Chem |  |  |  |
|  | PFS | mu = 2.28983, sigma =1.3453 | Log-normal |
|  | OS | mu = 3.062368, sigma =1.349783 | Log-normal |
| Nivo-Chem |  |  |  |
|  | PFS | scale = 13.1632, shape =1.47553 | Log-logistic |
|  | OS | scale = 27.70658, shape =1.49626 | Log-logistic |
| Suge-Chem |  |  |  |
|  | PFS | mu = 2.61114, sigma =1.12398 | Log-normal |
|  | OS | mu = 3.66768, sigma =1.41042 | Log-normal |
| Tisl-Chem |  |  |  |
|  | PFS | mu = 2.72356, sigma =1.31104 | Log-normal |
|  | OS | mu = 3.59854, sigma =1.24171 | Log-normal |
| Sint-Chem |  |  |  |
|  | PFS | mu = 2.59127, sigma =1.03309 | Log-normal |
|  | OS | mu = 3.57763, sigma =1.20321 | Log-normal |
| Tori-Chem |  |  |  |
|  | PFS | mu = 2.73882, sigma =1.08955 | Log-normal |
|  | OS | mu = 4.01572, sigma =1.45007 | Log-normal |
| Serp-Chem |  |  |  |
|  | PFS | mu = 2.82986, sigma =1.51143 | Log-normal |
|  | OS | mu = 3.704828, sigma =1.328379 | Log-normal |
| Chem |  |  |  |
|  | PFS | mu = 2.073318, sigma =0.96411 | Log-normal |
|  | OS | mu = 3.136252, sigma =1.252281 | Log-normal |

**Table S7 Treatment strategies compared in the model**

| **Strategy** | **1-4 cycle** | **4-35 cycle (two years）** | **2 years-10 years** |
| --- | --- | --- | --- |
| Tori-Chem | toripalimab (240 mg) + pemetrexed (500 mg/m2) + cisplatin (75 mg/m2) or carboplatin (AUC, 5 mg/mL/min) | toripalimab (240 mg) + pemetrexed (500 mg/m2) | pemetrexed (500 mg/m2) |
| Sint-Chem | sintilimab (200 mg) + pemetrexed (500 mg/m2) + cisplatin (75 mg/m2) or carboplatin (AUC, 5 mg/mL/min) | sintilimab (200 mg) + pemetrexed (500 mg/m2) | pemetrexed (500 mg/m2) |
| Carm-Chem | camrelizumab (200 mg) + pemetrexed (500 mg/m2) + cisplatin (75 mg/m2) or carboplatin (AUC, 5 mg/mL/min) | camrelizumab (200 mg) + pemetrexed (500 mg/m2) | pemetrexed (500 mg/m2) |
| Tisl-Chem | tislelizumab (200 mg) + pemetrexed (500 mg/m2) + cisplatin (75 mg/m2) or carboplatin (AUC, 5 mg/mL/min) | tislelizumab (200 mg) + pemetrexed (500 mg/m2) | pemetrexed (500 mg/m2) |
| Suge-Chem | sugemalimab (1200 mg) + pemetrexed (500 mg/m2) + cisplatin (75 mg/m2) or carboplatin (AUC, 5 mg/mL/min) | sugemalimab (1200 mg) + pemetrexed (500 mg/m2) | pemetrexed (500 mg/m2) |
| Serp-Chem | serplulimab (300 mg) + pemetrexed (500 mg/m2) + cisplatin (75 mg/m2) or carboplatin (AUC, 5 mg/mL/min) | serplulimab (300 mg) + pemetrexed (500 mg/m2) | pemetrexed (500 mg/m2) |
| Pemb-Chem | Pembrolizumab (200 mg) + pemetrexed (500 mg/m2) + cisplatin (75 mg/m2) or carboplatin (AUC, 5 mg/mL/min) | Pembrolizumab (200 mg) + pemetrexed (500 mg/m2) | pemetrexed (500 mg/m2) |
| Nivo-Chem | nivolumab (360 mg) + pemetrexed (500 mg/m2) + cisplatin (75 mg/m2) or carboplatin (AUC, 5 mg/mL/min) | nivolumab (360 mg) + pemetrexed (500 mg/m2) | pemetrexed (500 mg/m2) |
| Atez-Chem | atezolizumab (1200 mg) + pemetrexed (500 mg/m2) + cisplatin (75 mg/m2) or carboplatin (AUC, 5 mg/mL/min) | atezolizumab (1200 mg) + pemetrexed (500 mg/m2) | pemetrexed (500 mg/m2) |
|  | atezolizumab (1200 mg) + nab-Paclitaxel (100 mg/m², qw) + cisplatin (75 mg/m2) or carboplatin (AUC, 5 mg/mL/min) |  |  |
| Durv-Chem | durvalumab (1500 mg) + pemetrexed (500 mg/m2) + cisplatin (75 mg/m2) or carboplatin (AUC, 5 mg/mL/min) | durvalumab (1500 mg), q4w | pemetrexed (500 mg/m2) |
| Chem | pemetrexed (500 mg/m2) + cisplatin (75 mg/m2) or carboplatin (AUC, 5 mg/mL/min) | pemetrexed (500 mg/m2) | pemetrexed (500 mg/m2) |

**Table S8 The cost and disutility of grade ≥3 adverse events**

| **Regimens** | **Type of AEs** | **Cost per event ($)** | **Disutility** | PD-1/PD-L1 inhibitors + Chemotherapy | | | Chemotherapy | | |
| --- | --- | --- | --- | --- | --- | --- | --- | --- | --- |
|  |  |  |  | % | AEs cost | AEs disutility | % | AEs cost | AEs disutility |
| Camr-Chem | |  |  |  | 65.02 | 0.00358 |  | 45.11 | 0.00224 |
|  | Neutrophil count decreased | 93.49 | 0.00413 | 39.51% |  |  | 30.92% |  |  |
|  | WBC decreased | 93.49 | 0.00413 | 20.00% |  |  | 14.49% |  |  |
|  | Lymphocyte count decreased | 0 | 0.00413 | 4.39% |  |  | 2.42% |  |  |
|  | Myelosuppression | 137.44 | 0.01377 | 6.83% |  |  | 1.93% |  |  |
| Atez-Chem (IMpower130) | |  |  |  | 114.51 | 0.00648 |  | 86.37 | 0.00472 |
|  | Neutropenia | 93.49 | 0.00413 | 32.14% |  |  | 28.02% |  |  |
|  | Anemia | 49.34 | 0.01033 | 29.18% |  |  | 20.26% |  |  |
|  | Neutrophil count decreased | 93.49 | 0.00413 | 12.05% |  |  | 8.19% |  |  |
|  | Thrombocytopenia | 266.13 | 0.00757 | 9.51% |  |  | 6.47% |  |  |
|  | Platelet count decreased | 266.13 | 0.00757 | 7.82% |  |  | 6.03% |  |  |
|  | Fatigue | 106.75 | 0.00101 | 6.13% |  |  | 6.03% |  |  |
|  | White blood cell count decreased | 93.49 | 0.00413 | 6.55% |  |  | 3.02% |  |  |
| Atez-Chem (IMpower132 from China) | |  |  |  | 110.52 | 0.00652 |  | 55.64 | 0.00369 |
|  | Decreased neutrophil count | 93.49 | 0.00413 | 50.00% |  |  | 25.93% |  |  |
|  | Decreased white blood cell count | 93.49 | 0.00413 | 21.95% |  |  | 12.35% |  |  |
|  | Decreased platelet count | 266.13 | 0.00757 | 7.32% |  |  | 1.23% |  |  |
|  | Anemia | 49.34 | 0.01033 | 15.85% |  |  | 9.88% |  |  |
|  | Leukopenia | 93.49 | 0.00413 | 2.44% |  |  | 0 |  |  |
|  | Neutropenia | 93.49 | 0.00413 | 2.44% |  |  | 3.70% |  |  |
|  | Hyponatremia | 133.5 | 0.01359 | 8.54% |  |  | 6.17% |  |  |
| Sint-Chem | |  |  |  | 41.51 | 0.00306 |  | 37.96 | 0.00323 |
|  | Anemia | 49.34 | 0.01033 | 15.04% |  |  | 19.08% |  |  |
|  | Decreased neutrophil count | 93.49 | 0.00413 | 36.47% |  |  | 30.53% |  |  |
| Durv-Chem | |  |  |  | 28.44 | 0.00231 |  | 34.05 | 0.00250 |
|  | Anemia | 49.34 | 0.01033 | 15.27% |  |  | 20.42% |  |  |
|  | Thrombocytopenia | 266.13 | 0.00757 | 4.49% |  |  | 5.11% |  |  |
|  | Neutrophil count decreased | 93.49 | 0.00413 | 7.19% |  |  | 7.51% |  |  |
|  | Leukopenia | 93.49 | 0.00413 | 2.40% |  |  | 3.60% |  |  |
| Suge-Chem | |  |  |  | 15.84 | 0.00093 |  | 16.64 | 0.00097 |
|  | Anemia | 49.34 | 0.01033 | 4.38% |  |  | 4.40% |  |  |
|  | Aspartate aminotransferase increased | 157.42 | 0.00727 | 0.63% |  |  | 1.26% |  |  |
|  | White blood cell count decreased | 93.49 | 0.00413 | 4.38% |  |  | 4.40% |  |  |
|  | Platelet count decreased | 266.13 | 0.00757 | 3.13% |  |  | 3.14% |  |  |
|  | Leukopenia | 93.49 | 0.00413 | 0.31% |  |  |  |  |  |
| Pemb-Chem | |  |  |  | 55.19 | 0.00338 |  | 43.36 | 0.00293 |
|  | Anemia | 49.34 | 0.01033 | 19.01% |  |  | 17.82% |  |  |
|  | Neutropenia | 93.49 | 0.00413 | 16.79% |  |  | 11.88% |  |  |
|  | Asthenia | 106.75 | 0.00101 | 6.67% |  |  | 3.47% |  |  |
|  | Thrombocytopenia | 266.13 | 0.00757 | 8.64% |  |  | 7.43% |  |  |
| Tisl-Chem | |  |  |  | 70.73 | 0.00437 |  | 28.99 | 0.00224 |
|  | Anemia | 49.34 | 0.01033 | 15.32% |  |  | 11.82% |  |  |
|  | Leukopenia | 93.49 | 0.00413 | 22.97% |  |  | 7.21% |  |  |
|  | Neutropenia | 93.49 | 0.00413 | 44.59% |  |  | 17.57% |  |  |
| Tori-Chem | |  |  |  | 60.53 | 0.00448 |  | 65.48 | 0.00512 |
|  | Anemia | 49.34 | 0.01033 | 29.87% |  |  | 35.90% |  |  |
|  | Thrombocytopenia | 266.13 | 0.00757 | 17.21% |  |  | 17.95% |  |  |
|  | Lymphopenia | 0 | 0.00413 | 2.27% |  |  | 1.28% |  |  |
| Nivo-Chem | |  |  |  | 34.93 | 0.00221 |  | 28.90 | 0.00207 |
|  | Anemia | 49.34 | 0.01033 | 10.40% |  |  | 11.59% |  |  |
|  | Neutropenia | 93.49 | 0.00413 | 8.53% |  |  | 5.66% |  |  |
|  | Decreased neutrophil count | 93.49 | 0.00413 | 7.20% |  |  | 6.47% |  |  |
|  | Thrombocytopenia | 266.13 | 0.00757 | 4.27% |  |  | 3.50% |  |  |
|  | Decreased white blood cell count | 93.49 | 0.00413 | 4.00% |  |  | 2.70% |  |  |
| Serp-Chem | |  |  |  | 112.53 | 0.0067 |  | 102.32 | 0.00564 |
|  | Anemia | 49.34 | 0.01033 | 22.43% |  |  | 19.62% |  |  |
|  | Decreased neutrophil count | 93.49 | 0.00413 | 44.39% |  |  | 35.89% |  |  |
|  | Decreased white blood cell count | 93.49 | 0.00413 | 22.90% |  |  | 19.62% |  |  |
|  | Platelet count decreased | 266.13 | 0.00757 | 14.49% |  |  | 15.31% |  |  |
|  | Lymphocyte count decreased | 0 | 0.00413 | 11.21% |  |  | 3.83% |  |  |
| Cemi-Chem | |  |  |  |  |  |  | 12.20 | 0.00102 |
|  | Anemia | 49.34 | 0.01033 |  |  |  | 6.54% |  |  |
|  | Neutropenia | 93.49 | 0.00413 |  |  |  | 5.88% |  |  |
|  | Thrombocytopenia | 266.13 | 0.00757 |  |  |  | 1.31% |  |  |
| Reti-Chem | |  |  |  |  |  |  | 33.07 | 0.00232 |
|  | Anemia | 49.34 | 0.01033 |  |  |  | 12.11% |  |  |
|  | Decreased neutrophil count | 93.49 | 0.00413 |  |  |  | 10.53% |  |  |
|  | Decreased white blood cell count | 93.49 | 0.00413 |  |  |  | 4.74% |  |  |
|  | Neutropenia | 93.49 | 0.00413 |  |  |  | 4.74% |  |  |
|  | Platelet count decreased | 266.13 | 0.00757 |  |  |  | 3.16% |  |  |
| Prol-Chem | |  |  |  |  |  |  | 80.92 | 0.00371 |
|  | Anemia | 49.34 | 0.01033 |  |  |  | 11.49% |  |  |
|  | Neutrophil count decreased | 93.49 | 0.00413 |  |  |  | 14.19% |  |  |
|  | Neutropenia | 93.49 | 0.00413 |  |  |  | 13.51% |  |  |
|  | White blood cell count decreased | 93.49 | 0.00413 |  |  |  | 9.46% |  |  |
|  | Asthenia | 106.75 | 0.00101 |  |  |  | 10.81% |  |  |
|  | Platelet count decreased | 266.13 | 0.00757 |  |  |  | 9.46% |  |  |
|  | Leukopenia | 93.49 | 0.00413 |  |  |  | 4.05% |  |  |

**Table S9 The details of including trails**

| study | Time | country | Disease stage | strategy | age  (mean) | sex  (male) | **Efficacy** | | | | **Safety** | | | | |  |
| --- | --- | --- | --- | --- | --- | --- | --- | --- | --- | --- | --- | --- | --- | --- | --- | --- |
|  |  |  |  |  |  |  | n | OS  HR (95%CI) | PFS HR (95%CI) | CR+PR | | n | Aes | grade≥3 AEs | discontinuation | |
| ORIENT-11 | August 23, 2018 and July 30, 2019 | China | III-IV | Sintilimab+ pemetrexed + cisplatin or carboplatin q3w | 61 | 204 | 266 | 0.65 (0.5-0.85) | 0.482 (0.363-0.643) | 138 | | 266 | 265 | 164 | 16 | |
|  |  |  |  | pemetrexed + cisplatin or carboplatin q3w | 61 | 99 | 131 |  |  | 39 | | 131 | 131 | 77 | 11 | |
| ASTRUM-002 | Nov 25, 2019 and June 15, 2022 | China | III-IV | serplulimab + pemetrexed + carboplatin q3w | 62 | 157 | 223 | 0.66 (0.52-0.83) | 0.54 (0.43-0.68) | 129 | | 214 | 212 | 145 | 33 | |
|  |  |  |  | pemetrexed + carboplatin q3w | 61 | 156 | 111 |  |  | 40 | | 209 | 205 | 118 | 15 | |
| CHOICE-01 | April 2, 2019 and August 5, 2020 | China | III-IV | Toripalimab + pemetrexed + cisplatin or carboplatin q3w | 62 | 115 | 162 | 0.49 (0.35-0.69) | 0.48 (0.35-0.66) |  | | 308 | 305 | 242 | 56 | |
|  |  |  |  | pemetrexed + cisplatin or carboplatin q3w | 60 | 64 | 83 |  |  |  | | 156 | 156 | 128 | 9 | |
| POD1UM | Sept 11, 2020, and March 14 | multiregional | IV | Retifanlimab + pemetrexed + cisplatin or carboplatin | 62.8 |  | 258 | 0.77 (0.58-1) | 0.67 (0.52-0.87) |  | | 389 | 369 | 238 | 80 | |
|  |  |  |  | pemetrexed + cisplatin or carboplatin q3w | 63 |  | 123 |  |  |  | | 190 | 183 | 103 | 27 | |
| POSEIDON | June 27, 2017, and September 19, 2018, | multiregional | IV | Durvalumab + pemetrexed+ cisplatin or carboplatin q3w | 64.5 |  | 203 | 0.81 (0.66-1.00) | 0.77 (0.61-0.96) | 90 | | 334 | 296 | 149 | 47 | |
|  |  |  |  | pemetrexed + cisplatin or carboplatin q3w | 64 |  | 211 |  |  | 50 | | 333 | 298 | 148 | 59 | |
| GEMSTONE-302 | Dec 13, 2018, and May 15, 2020 | China | IV | sugemalimab + carboplatin and pemetrexed q3w | 62 |  | 191 | 0.72 (0.53-0.98) | 0.57 (0.44-0.75) | 112 | | 320 | 317 | 182 | 57 | |
|  |  |  |  | pemetrexed + carboplatin q3w | 64 |  | 96 |  |  | 35 | | 159 | 153 | 91 | 18 | |
| EMPOWER-Lung 3 | 17 June 2019 and 30 September 2020, | multiregional | III-IV | cemiplimab + platinum-doublet chemotherapy | 63 |  | 179 | 0.64 (0.47-0.88) | 0.53 (0.39-0.71) | 74 | | 312 | 301 | 152 | 19 | |
|  |  |  |  | pemetrexed + cisplatin or carboplatin q3w | 63 |  | 87 |  |  | 16 | | 153 | 145 | 50 | 7 | |
| KEYNOTE-189 | February 26, 2016, and March 6, 2017 | multiregional | IV | pembrolizumab + pemetrexed + cisplatin or carboplatin q3w | 65 | 254 | 410 | 0.6 (0.5-0.72) | 0.5 (0.42-0.6) | 198 | | 405 | 377 | 212 | 145 | |
|  |  |  |  | pemetrexed + cisplatin or carboplatin q3w | 63.5 | 109 | 206 |  |  | 41 | | 202 | 183 | 85 | 35 | |
| CheckMate 227 Part II | August 2015 through November 2016 | multiregional | IV | Nivolumab + pemetrexed + cisplatin or carboplatin q3w | 64 | 293 | 270 | 0.86 (0.69-1.08) | 0.67 (0.55-0.82) | 130 | | 375 | 318 | 168 | 73 | |
|  |  |  |  | pemetrexed + cisplatin or carboplatin q3w | 64 | 385 | 273 |  |  | 80 | | 371 | 291 | 131 | 31 | |
| IMpower130 | April 16, 2015, and February 13, 2017 | multiregional | IV | Atezolizumab + Carboplatin + nab-Paclitaxel q3w | 64 | 277 | 447 | 0.79 (0.64-0.98) | 0.64 (0.54-0.77) | 220 | | 473 | 455 | 354 | 125 | |
|  |  |  |  | Carboplatin + nab-Paclitaxel | 65 | 138 | 226 |  |  | 72 | | 232 | 215 | 141 | 51 | |
| IMpower132 | April 7, 2016, and May 31, 2017. | multiregional | IV | Atezolizumab + pemetrexed+ cisplatin or carboplatin q3w | 64 | 192 | 292 | 0.86 (0.71-1.06) | 0.6 (0.49-0.72) | 137 | | 291 | 266 | 170 | 83 | |
|  |  |  |  | pemetrexed + cisplatin or carboplatin q3w | 63 | 192 | 286 |  |  | 91 | | 274 | 240 | 118 | 50 | |
| RATIONALE 304 | July 23, 2018, and July 31, 2019 | China | III- IV | Tislelizumab + pemetrexed + cisplatin or carboplatin q3w | 66 | 168 | 214 | 0.87 (0.65-1.17) | 0.61 (0.46-0.82) | 113 | | 222 | 222 | 154 | 68 | |
|  |  |  |  | pemetrexed + cisplatin or carboplatin q3w | 61 | 79 | 210 |  |  | 58 | | 110 | 109 | 62 | 11 | |
| CameL | May 12, 2017, and June 6, 2018 | China | IIIB–IV | Camrelizumab + pemetrexed+ carboplatin q3w | 59 | 146 | 205 | 0.74 (0.58-0.93) | 0.55 (0.44-0.68) | 113 | | 205 | 204 | 146 |  | |
|  |  |  |  | pemetrexed + carboplatin q3w | 61 | 149 | 207 |  |  | 68 | | 207 | 199 | 102 |  | |
| DOMAJOR | September 13, 2019, and November 30, 2022 | multiregional | IV | Prolgolimab + pemetrexed + carboplatin q3w | 62 | 95 | 143 | 0.51 (0.35-0.73) | 0.65 (0.49-0.85) | 72 | | 143 | 139 | 81 | 22 | |
|  |  |  |  | Pemetrexed + carboplatin q3w | 62 | 107 | 149 |  |  | 41 | | 148 | 132 | 55 | 16 | |

**Table S10 The score of the surface under the cumulative ranking in effectiveness and safety**

| Strategy | **Efficacy (rank (SUCRA))** | | | **Safety (rank (SUCRA))** | | |
| --- | --- | --- | --- | --- | --- | --- |
|  | overall survival | progression-free survival | objective response rate | adverse events | grade≥3 adverse events | treatment discontinuation due to adverse events |
| Sint-Chem | **1 (0.823)** | 5 (0.656) | 6 (0.523) | 10 (0.313) | 10 (0.288) | 13 (0.081) |
| Tori-Chem | 2 (0.819) | **1 (0.922)** |  | **14 (0.149)** | **14 (0.072)** | 2 (0.882) |
| Pemb-Chem | 3 (0.802) | 3 (0.775) | **1 (0.897)** | 9 (0.461) | 7 (0.547) | 3 (0.766) |
| Cemi-Chem | 4 (0.688) | 4 (0.670) | 2 (0.713) | 8 (0.490) | 3 (0.772) | 10 (0.373) |
| Serp-Chem | 5 (0.670) | 6 (0.639) | 3 (0.682) | 5 (0.580) | 6 (0.611) | 5 (0.689) |
| Camr-Chem | 6 (0.640) | 8 (0.463) | 7 (0.509) | 1 (0.873) | 1 (0.934) | 10 (0.373) |
| Suge-Chem | 7 (0.574) | 7 (0.503) | 8 (0.493) | 3 (0.789) | 12 (0.178) | 6 (0.497) |
| Tisl-Chem | 8 (0.462) | 13 (0.228) | 9 (0.476) | 4 (0.762) | 5 (0.676) | 1 (0.917) |
| Atez-Chem | 9 (0.413) | 11 (0.279) | 11 (0.226) | 6 (0.560) | 4 (0.757) | 9 (0.413) |
| Prol-Chem | 10 (0.359) | 2 (0.889) | 4 (0.575) | 2 (0.808) | 2 (0.849) | 8 (0.424) |
| Reti-Chem | 11 (0.310) | 9 (0.401) |  | 13 (0.182) | 9 (0.430) | 7 (0.445) |
| Nivo-Chem | 12 (0.296) | 12 (0.231) | 10 (0.374) | 7 (0.515) | 8 (0.530) | 4 (0.762) |
| Durv-Chem | 13 (0.142) | 10 (0.318) | 5 (0.533) | 12 (0.244) | 11 (0.187) | **14 (0.073)** |
| Chem | 14 (0.001) | 14 (0.027) | 12 (0.000) | 11 (0.274) | 13 (0.169) | 12 (0.180) |

**Table S11 Cost-effectiveness of PD-1/PD-L1 inhibitor with chemotherapy in non-squamous NSCLC patients base on life-year**

| **Strategy** | **Cost ($)** | **Effect (LYs)** | **Incremental effect (Cost/LY)** | | | | |
| --- | --- | --- | --- | --- | --- | --- | --- |
|  |  |  | **vs. Chem** | **vs. Sint_Chem** | **vs. Tisl_Chem** | **vs. Camr_Chem** | **vs. Tori_Chem** |
| **Chem** | 20,766.57 | 1.437 | - |  |  |  |  |
| **Sint_Chem** | 31,634.39 | 1.972 | 20,315.01 | **-** |  |  |  |
| **Tisl_Chem** | 33,720.70 | 2.109 | 19,278.47 | 15,230.43 | - |  |  |
| **Camr_Chem** | 36,365.21 | 2.313 | 17,813.78 | 13,886.20 | 12,982.25 | - |  |
| **Tori_Chem** | 39,232.33 | 2.583 | 16,120.12 | 12,444.52 | 11,638.67 | 10,624.47 | - |
| **Serp_Chem** | 58,177.27 | 2.335 | 41694.4 | 73,263.37 | 108,545.83 | 1,009,397.01 | undominated |
| **Suge_Chem** | 85,538.95 | 2.180 | 87,203.58 | 259,397.23 | 731,648.17 | undominated | undominated |
| **Atez_Chem** | 85,746.44 | 1.582 | 449,236.89 | undominated | undominated | undominated | undominated |
| **Nivo_Chem** | 94,304.63 | 1.694 | 286,475.83 | undominated | undominated | undominated | undominated |
| **Durv_Chem** | 104,369.24 | 1.530 | 905,496.38 | undominated | undominated | undominated | undominated |
| **Pemb_Chem** | 106,801.78 | 1.912 | 181,400.58 | undominated | undominated | undominated | undominated |
